# Supplementary material for: The architecture of Cidec-mediated interfaces between lipid droplets
Source: Cell Rep. 2023 Feb 16;42(2):112107. doi: 10.1016/j.celrep.2023.112107 (PMC9989828; doi:10.1016/j.celrep.2023.112107)
Supplement: Document S1. Figures S1–S5 [file mmc1.pdf]

**Cell Reports, Volume *42***

## Supplemental information

# The architecture of Cidec-mediated interfaces between lipid droplets

**Iva Ganeva, Koini Lim, Jerome Boulanger, Patrick C. Hoffmann, Olivia Muriel, Alicia C. Borgeaud, Wim J.H. Hagen, David B. Savage, and Wanda Kukulski**

## Supplemental Figures and Legends

Figure S1

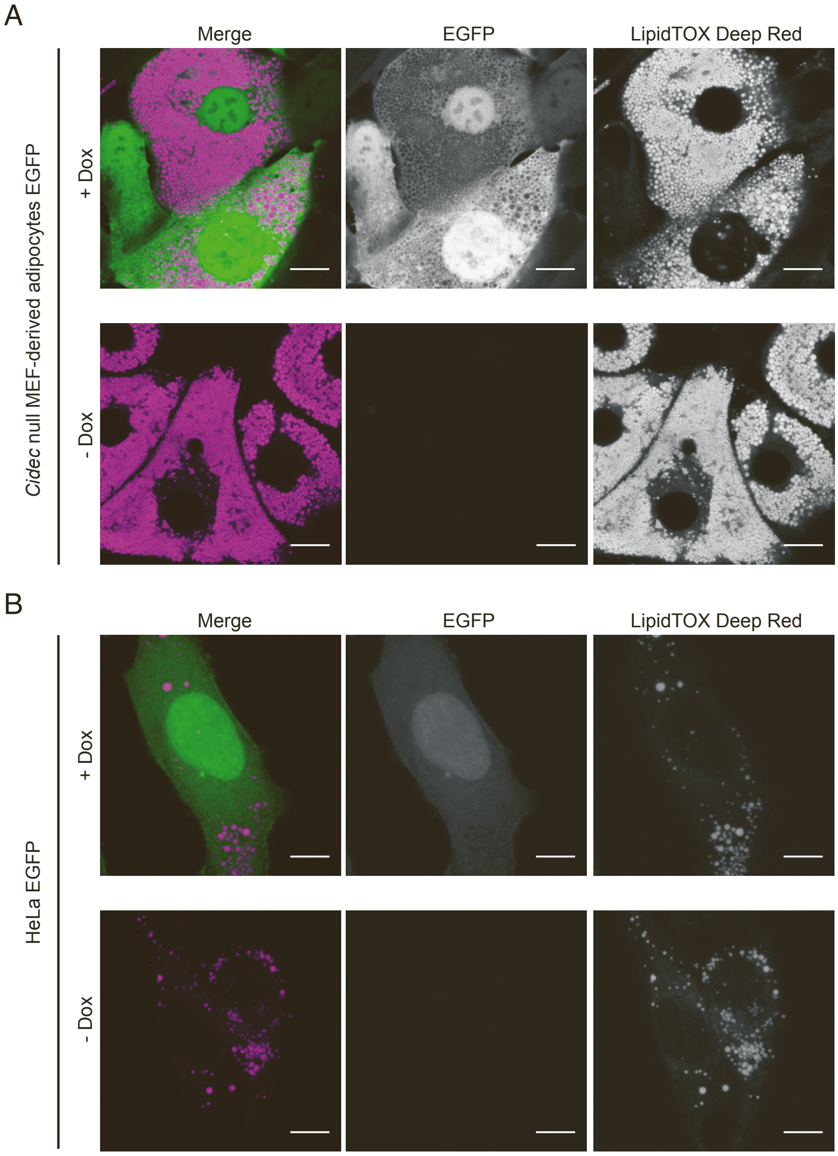


<u>Supplemental Figure S1: Expression of cytosolic EGFP.</u> Related to Figure 1. **A)** Fluorescence microscopy (FM) of fixed *Cidec* null MEF-derived adipocytes inducibly expressing cytosolic EGFP (green). Lipid droplets (LDs) were stained with LipidTOX Deep Red dye (magenta). *Cidec* null MEF-derived adipocytes were induced with Doxycycline (upper panels, + Dox) or not induced (lower panels, -Dox) for expression of cytosolic EGFP throughout the course of differentiation. **B)** FM of fixed HeLa cells inducibly expressing cytosolic EGFP. LDs were stained with LipidTOX Deep Red. HeLa cells were induced with Doxycycline (upper panels, +Dox) or not induced (lower panels, -Dox) for expression of cytosolic EGFP. Images in B were acquired on fixed HeLa cells 24 hours after the corresponding treatment (+Dox/ -Dox). Scale bars: 10 $\mu$m.

Figure S2

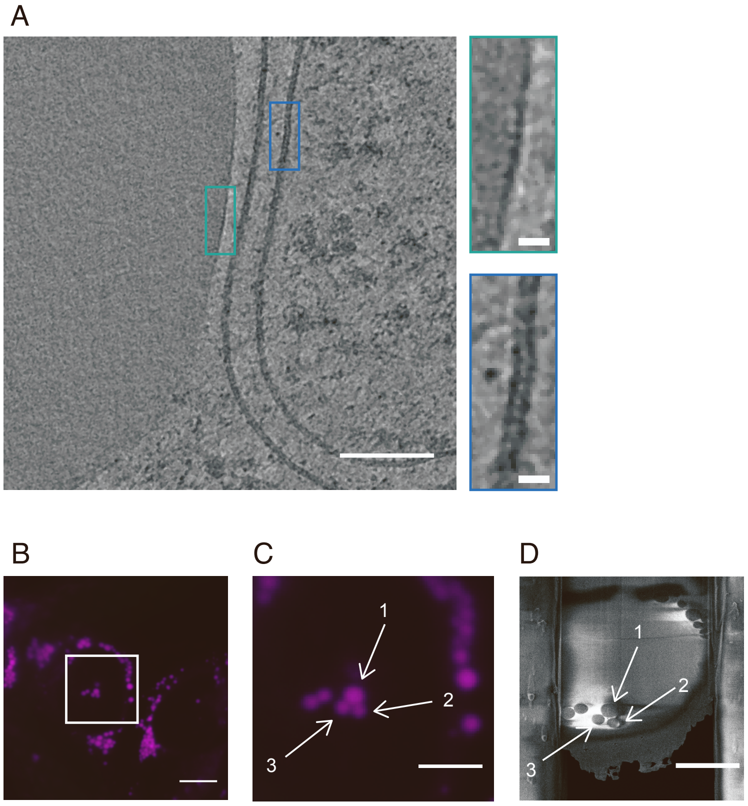


Supplemental Figure S2: Cryo-correlative light and electron microscopy (cryo-CLEM) of HeLa cells expressing Cidec constructs. Related to Figures 2 and 3. **A)** Comparison of the appearance of a monolayer with the appearance of a bilayer. Virtual slice through an electron cryo-tomogram of a HeLa cell inducibly expressing Cidec-EGFP. Green box shows a magnified view of a monolayer, blue box shows a magnified view of a bilayer. **B and C)** Cryo-FM of plunge-frozen HeLa cells induced for expression of untagged Cidec, grown on a cryo-EM grid. Region for cryo-focused ion beam (cryo-FIB) milling was chosen based on LD size, indicating enlargement. **C)** Magnified view of the region shown in the white box in B. White arrows indicate LDs. **D)** Cryo-scanning electron microscopy (cryo-SEM) overview image of a lamella generated from the HeLa cell shown in B and C, thinned by cryo-FIB milling. Interacting LDs identified by cryo-FM are visible in the resulting lamella (white arrows). Scale bars: 100 nm in A (large image), 10 nm in A (magnified views), 10 $\mu$m in B, 5 $\mu$m in C and D.

Figure S3

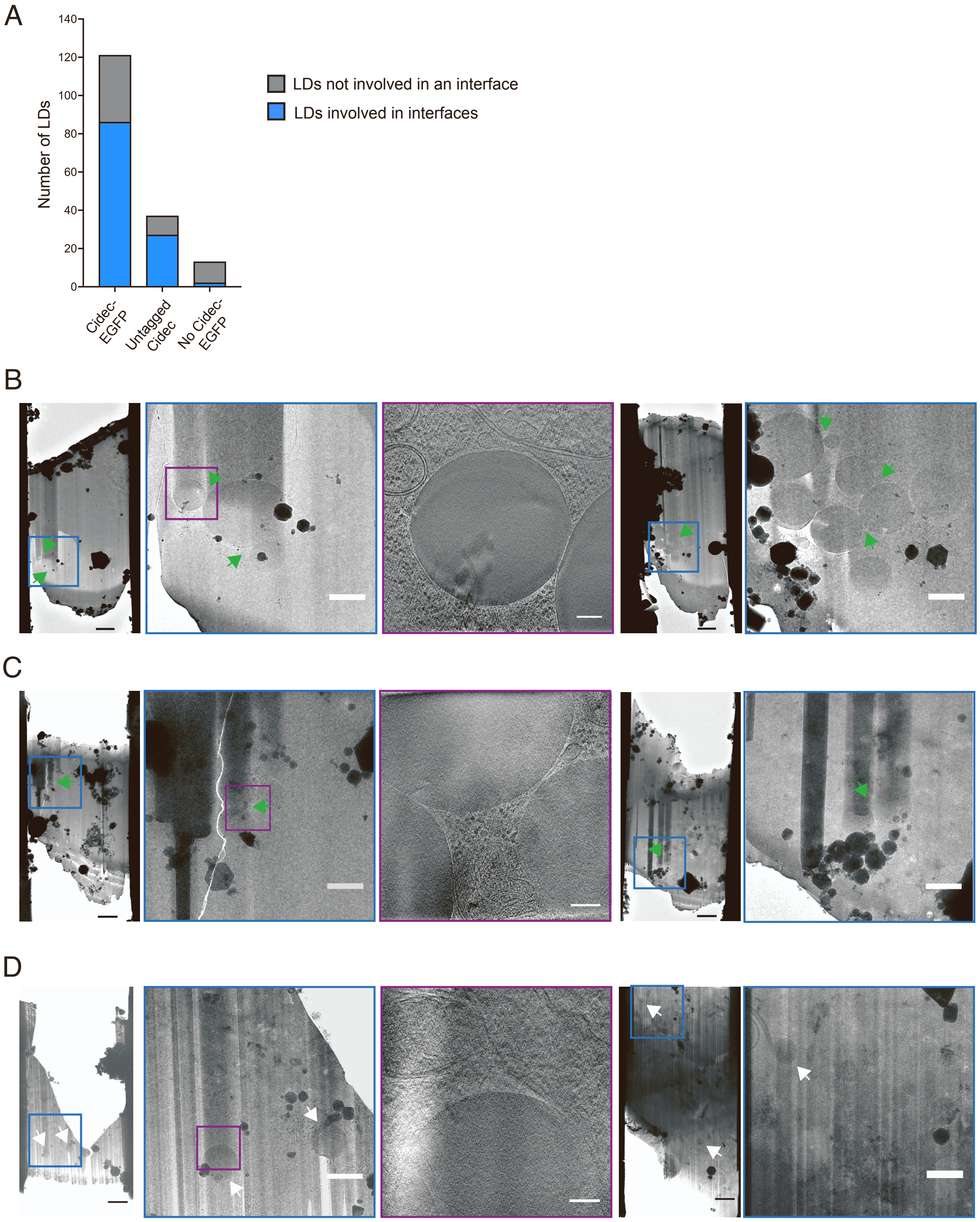


Supplemental Figure S3: Proximity of LDs to each other in cells expressing Cidec-EGFP, untagged Cidec, and non-induced cells. Related to Figures 2 and 3. HeLa cells were fed with oleic acid and either induced with Doxycycline for Cidec-EGFP or Cidec expression, or not induced for Cidec-EGFP expression by not adding Doxycycline. Plunge frozen cells were subjected to cryo-FIB milling and imaged by cryo-EM. **A)** Number of LDs visualised by 2D cryo-EM imaging of lamellae of cells expressing

Cidec-EGFP, untagged Cidec, or not expressing Cidec-EGFP. The LDs were classified into two groups, based on whether they were in contact with at least one other LD or not. 71% of the LDs (n=121) in lamellae of Cidec-EGFP expressing cells and 73% of LDs (n=37) in lamellae of Cidec expressing cells were forming interfaces in contrast to 15% of LDs (n=13) in lamellae of cells not expressing Cidec-EGFP (P=0.0002 for Chi-square contingency test). Note that all lamellae containing LDs of which cryo-EM overview maps were acquired were used for this analysis, including lamellae which were not subjected to electron cryo-tomography (cryo-ET). **B - D)** HeLa cells expressing Cidec-EGFP (B), untagged Cidec (C) or not induced for expression of Cidec-EGFP (D) were vitrified, screened for LD-LD interfaces by cryo-FM, thinned by cryo-FIB milling and imaged by cryo-EM. The first and fourth panels from the left represent overview cryo-EM images of lamellae. Second and fifth panels correspond to a magnified image of the areas in blue boxes. Third panels represent virtual slices through tomograms acquired at areas where LDs are in close proximity indicated by the purple boxes in the second panel (B and C) or of an individual LD (D). Green arrows point to LD-LD interfaces. White arrows indicate individual LDs. Note that the second panel in B is from the same image as the fourth panel in Figure 1C. Scale bars 2.5 $\mu$m (first and fourth panels), 1 $\mu$m (second and fifth panels), and 150 nm (third panels).

Figure S4

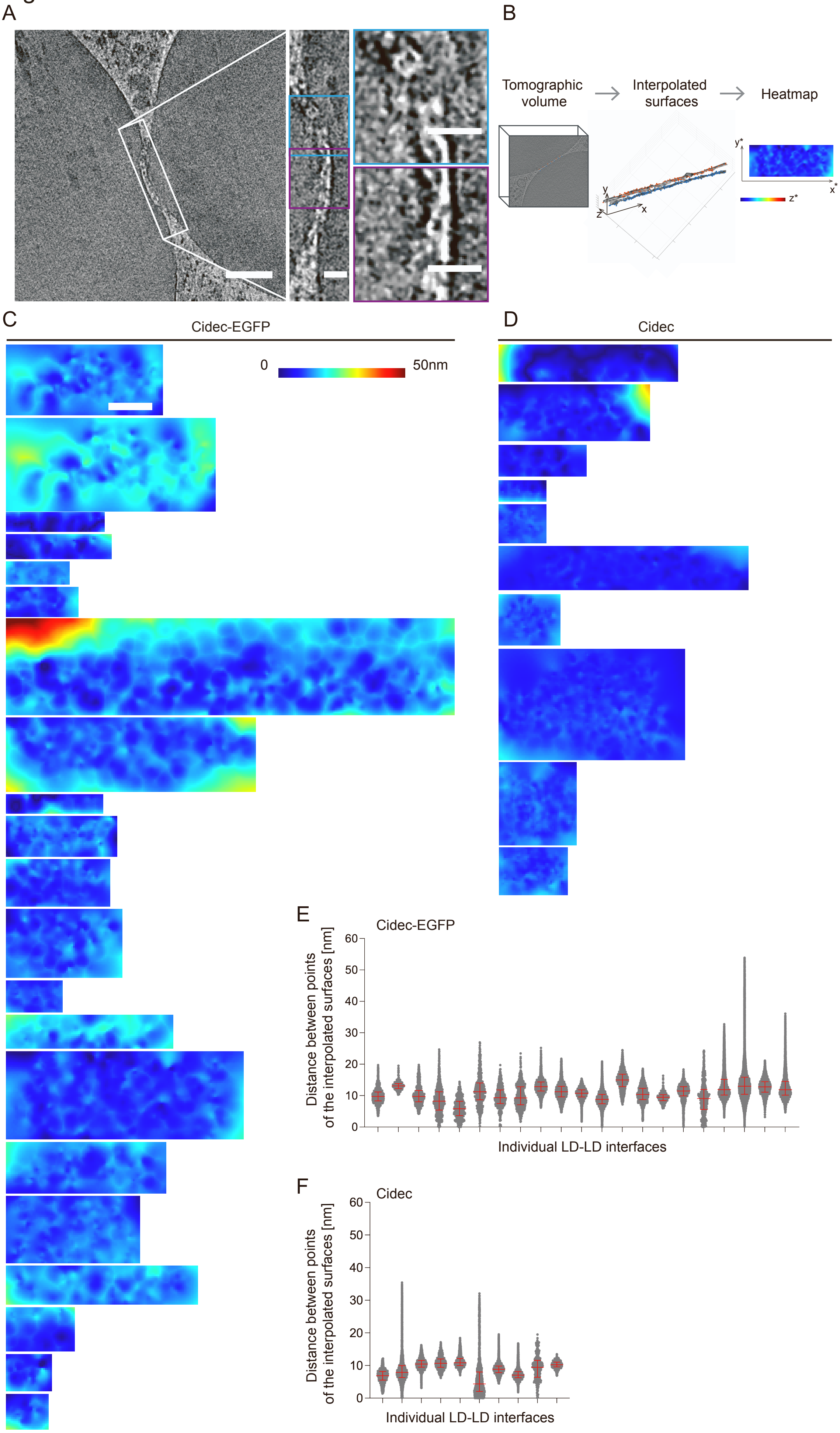

A
B
Tomographic volume
Interpolated surfaces
Heatmap
y
x
z
y*
x*
z*
C
Cidec-EGFP
0
50nm
D
Cidec
E
Cidec-EGFP
Distance between points of the interpolated surfaces [nm]
60
50
40
30
20
10
0
Individual LD-LD interfaces
F
Cidec
Distance between points of the interpolated surfaces [nm]
60
50
40
30
20
10
0
Individual LD-LD interfaces

Supplemental Figure S4: Details of LD-LD interfaces. Related to Figures 2 and 3. **A)** Virtual slices through a tomogram acquired at an area where LDs are in close proximity. HeLa cells inducibly expressing Cidec-EGFP were vitrified, screened for LD interfaces by cryo-FM, thinned by cryo-FIB milling and imaged by cryo-ET. The shape of the LDs is deformed where two LDs are closely apposed. The morphology displayed here shows a protrusion of one LD into the other LD, resulting in an indentation. The image is the same as shown in Figure 2C. The degree of deformation varies along the interface (white rectangle, magnified in second image): some areas display large deformation of the monolayers (blue square) and others slight waviness (purple square). Scale bars: 100 nm in large image (left panel) and 25 nm in magnified views. **B)** Schematic overview of distance measurements at LD interfaces. Points were picked manually along both monolayers in virtual tomographic slices. The monolayers were thereby traced through the entire tomographic volume (first panel). Based on the manually picked points, interpolated surfaces are computed as 2 nm-spaced regular grid points (middle panel). The distances between these grid points are represented as a heatmap, with x, y corresponding to the position of the regular grid (third panel). **C)** and **D)** Heat maps (generated as explained in B) illustrating the variability of distances between the monolayers at interface formed by Cidec-EGFP (C) and by untagged Cidec (D). Each panel corresponds to one individual interface. The scale bar in the top left panel corresponds to 50 nm and applies to all panels. The heat map legend also applies to all panels; dark blue corresponds to an inter-monolayer distance of 0 nm and dark red corresponds to an inter-monolayer distance of 50 nm. **E)** and **F)** The distances measured between the regularly spaced points of the interpolated surfaces plotted as scatter plots for each individual interface. The red lines correspond to medians and interquartile range. The medians are plotted in Figure 3F. **E)** Interfaces between LDs in cells expressing Cidec-EGFP. **F)** Interfaces between LDs in cells expressing untagged Cidec.

Figure S5

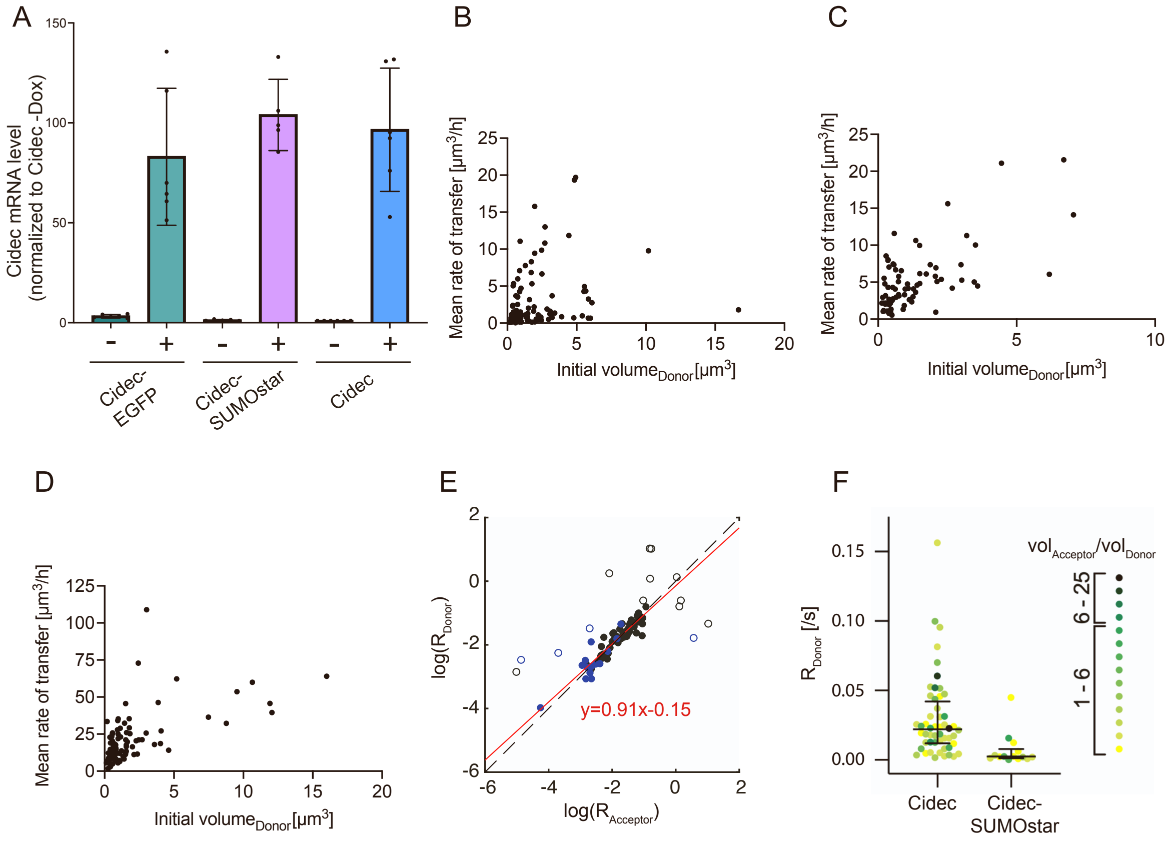


<u>Supplemental Figure S5: Characterisation of Cidec constructs.</u> Related to Figures 4 and 5. **A)** HeLa cell lines were induced with Doxycycline for expression of Cidec constructs in the presence of 200 μM oleic acid for 24 hours. Comparable levels of *Cidec* transcripts were achieved by using 0.5 μg/mL of Doxycycline in Cidec-EGFP and untagged Cidec HeLa cell lines and 2.0 μg/mL of Doxycycline in the Cidec-SUMOstar HeLa cell line. mRNA levels were normalized to the level of untagged Cidec HeLa cell line without Doxycycline treatment. The plot shows mean Cidec mRNA expression levels with SD from at least 5 independent experiments. **B-D)** The mean rates of transfer (same data as shown in Figure 4D) plotted against the initial volume of the donor LD measured at the start of the event, for Cidec-EGFP (B), Cidec-SUMOstar (C) and untagged Cidec (D). **E)** Detection of outliers. Log10 of R-values, calculated from exponential fits as shown in Figure 5C, D and E, of acceptor LD are plotted against log10 of R-values of the corresponding donor LD. Cidec: black circles. Cidec-SUMOstar: blue circles. Red equation describes regression line (shown in red). Black dashed line indicates y=x. Pairs differing by more than 0.4 (empty circles) were excluded from further analysis (for details see text and STAR Methods). **F)** $R_{Donor}$ values for untagged Cidec (n=52) and Cidec-SUMOstar (n=14). This is the same data

as shown in Figure 5F, except that each data point, corresponding to one event, is color-coded according to the starting ratio of acceptor volume to donor volume. Black bars correspond to median and interquartile range (Cidec: n=52; median: 0.022 /s, Cidec-SUMOstar: n=14; median: 0.002 /s. P<0.0001).
