# Supplementary material for: The architecture of Cidec-mediated interfaces between lipid droplets
Source: Cell Rep. 2023 Feb 16;42(2):112107. doi: 10.1016/j.celrep.2023.112107 (PMC9989828; doi:10.1016/j.celrep.2023.112107)
Supplement: Document S2. Article plus supplemental information [file mmc12.pdf]

Cell Reports

Article

The architecture of Cidec-mediated interfaces between lipid droplets

Graphical abstract

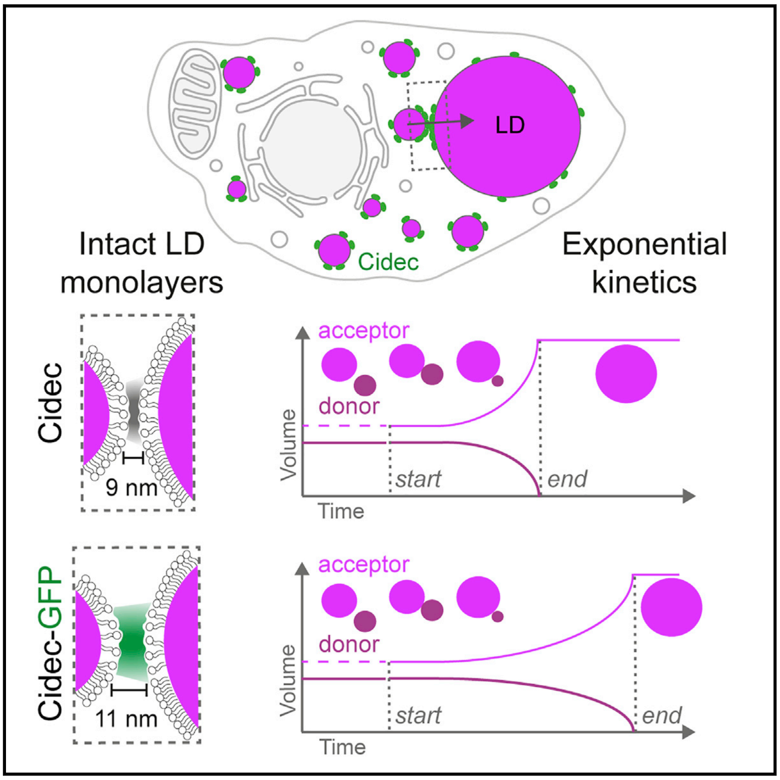

Authors

Iva Ganeva, Koini Lim,
Jerome Boulanger, ..., Wim J.H. Hagen,
David B. Savage, Wanda Kukulski

Correspondence

dbs23@medschl.cam.ac.uk (D.B.S.),
wanda.kukulski@unibe.ch (W.K.)

In brief

Cidec is required for formation of unilocular lipid droplets in white adipocytes, but exactly how it does so remains unclear. Ganeva et al. visualize Cidec-mediated droplet growth and observe that lipid transfer between droplets accelerates while interacting lipid droplets remain distinct entities, suggesting that Cidec facilitates pressure-driven lipid transfer.

Highlights

- Cidec mediates close (≈10 nm) apposition of lipid droplets for neutral lipid transfer
- Tagging Cidec increases the distance between droplets and slows lipid transfer
- Cidec-mediated transfer of neutral lipids accelerates with exponential kinetics

Ganeva et al., 2023, Cell Reports *42*, 112107
February 28, 2023 © 2023 MRC Laboratory of Molecular Biology.
https://doi.org/10.1016/j.celrep.2023.112107

CellPress

Cell Reports

CellPress
OPEN ACCESS

Article

The architecture of Cidec-mediated interfaces between lipid droplets

Iva Ganeva,[1,2,9] Koini Lim,[3,9] Jerome Boulanger,[1] Patrick C. Hoffmann,[1,7] Olivia Muriel,[4,5] Alicia C. Borgeaud,[2] Wim J.H. Hagen,[6] David B. Savage,[3,8,*] and Wanda Kukulski[1,2,8,10,*]
[1]MRC Laboratory of Molecular Biology, Francis Crick Avenue, Cambridge CB2 0QH, UK
[2]Institute of Biochemistry and Molecular Medicine, University of Bern, Bühlstrasse 28, 3012 Bern, Switzerland
[3]Metabolic Research Laboratories, Wellcome Trust-Medical Research Council Institute of Metabolic Science, University of Cambridge, Cambridge CB2 0QQ, UK
[4]Electron Microscopy Facility, University of Lausanne, Biophore Building, 1015 Lausanne, Switzerland
[5]Department of Fundamental Microbiology, Faculty of Biology and Medicine, University of Lausanne, Biophore Building, 1015 Lausanne, Switzerland
[6]Structural and Computational Biology Unit, European Molecular Biology Laboratory, Meyerhofstrasse 1, 69117 Heidelberg, Germany
[7]Present address: Department of Molecular Sociology, Max Planck Institute of Biophysics, Max-von-Laue-Strasse 3, 60438 Frankfurt am Main, Germany
[8]Senior author
[9]These authors contributed equally
[10]Lead contact
*Correspondence: dbs23@medschl.cam.ac.uk (D.B.S.), wanda.kukulski@unibe.ch (W.K.)
https://doi.org/10.1016/j.celrep.2023.112107

SUMMARY

Lipid droplets (LDs) are intracellular organelles responsible for storing surplus energy as neutral lipids. Their size and number vary enormously. In white adipocytes, LDs can reach 100 μm in diameter, occupying >90% of the cell. Cidec, which is strictly required for the formation of large LDs, is concentrated at interfaces between adjacent LDs and facilitates directional flux of neutral lipids from the smaller to the larger LD. The mechanism of lipid transfer is unclear, in part because the architecture of interfaces between LDs remains elusive. Here we visualize interfaces between LDs by electron cryo-tomography and analyze the kinetics of lipid transfer by quantitative live fluorescence microscopy. We show that transfer occurs through closely apposed monolayers, is slowed down by increasing the distance between the monolayers, and follows exponential kinetics. Our data corroborate the notion that Cidec facilitates pressure-driven transfer of neutral lipids through two "leaky" monolayers between LDs.

INTRODUCTION

White adipocytes, which are responsible for regulated fat storage and release in vertebrates, feature a single large lipid droplet (LD) filling up almost their entire cytoplasm. This distinctive morphology is a key factor in optimizing energy storage capacity and reducing the relative surface area, thereby enabling tight regulation of lipid storage and release from the droplet.[1] Excessive or deficient lipid storage is a hallmark of several serious and highly prevalent metabolic diseases such as diabetes, non-alcoholic fatty liver disease, and atherosclerosis. Both human and mouse loss-of-function genetic data suggest that Cidec is strictly required for the formation of unilocular white adipocytes.[2–5] Specifically, many white adipocytes in a patient with a nonsense *CIDEC* mutation (E186X) were shown to be multilocular,[4] and, in *Cidec* null mice, all white adipocytes were multilocular.[2,3] Gain-of-function studies involving expression of Cidec-GFP in heterologous cells also showed that Cidec consistently enlarged LDs, although the droplets remain multilocular in all these instances.[6–10] These studies also showed that Cidec localized on the surface of LDs and accumulated at interfaces between LDs, where it facilitated net transfer of neutral lipid from the smaller to the larger LD. Barneda et al. extended these observations by suggesting that an amphipathic helix of the homologous protein Cidea was involved in recruiting the protein to the LD surface, in perturbing the phospholipid monolayer, and in facilitating neutral lipid transfer across the interface.[9]

RESULTS

To emulate the requirement for Cidec, we first used *Cidec* null mouse embryonic fibroblast (MEF)-derived adipocytes[2] and expressed Cidec-EGFP during adipogenic differentiation using a lentiviral Tet-ON inducible system. Upon expression, Cidec-EGFP was targeted to the surface of LDs and enriched at interfaces between LDs (Figure 1A). The volume of LDs increased approximately 30-fold compared with LDs in cells that were not induced (Figure 1A) (median −Dox 0.86 μm^3, median +Dox 31.79 μm^3, $p < 0.0001$, $n = 75$ cells per condition). Expression of cytosolic EGFP had no effect on LDs (Figure S1A). These

© 2023 MRC Laboratory of Molecular Biology.
This is an open access article under the CC BY license (http://creativecommons.org/licenses/by/4.0/).

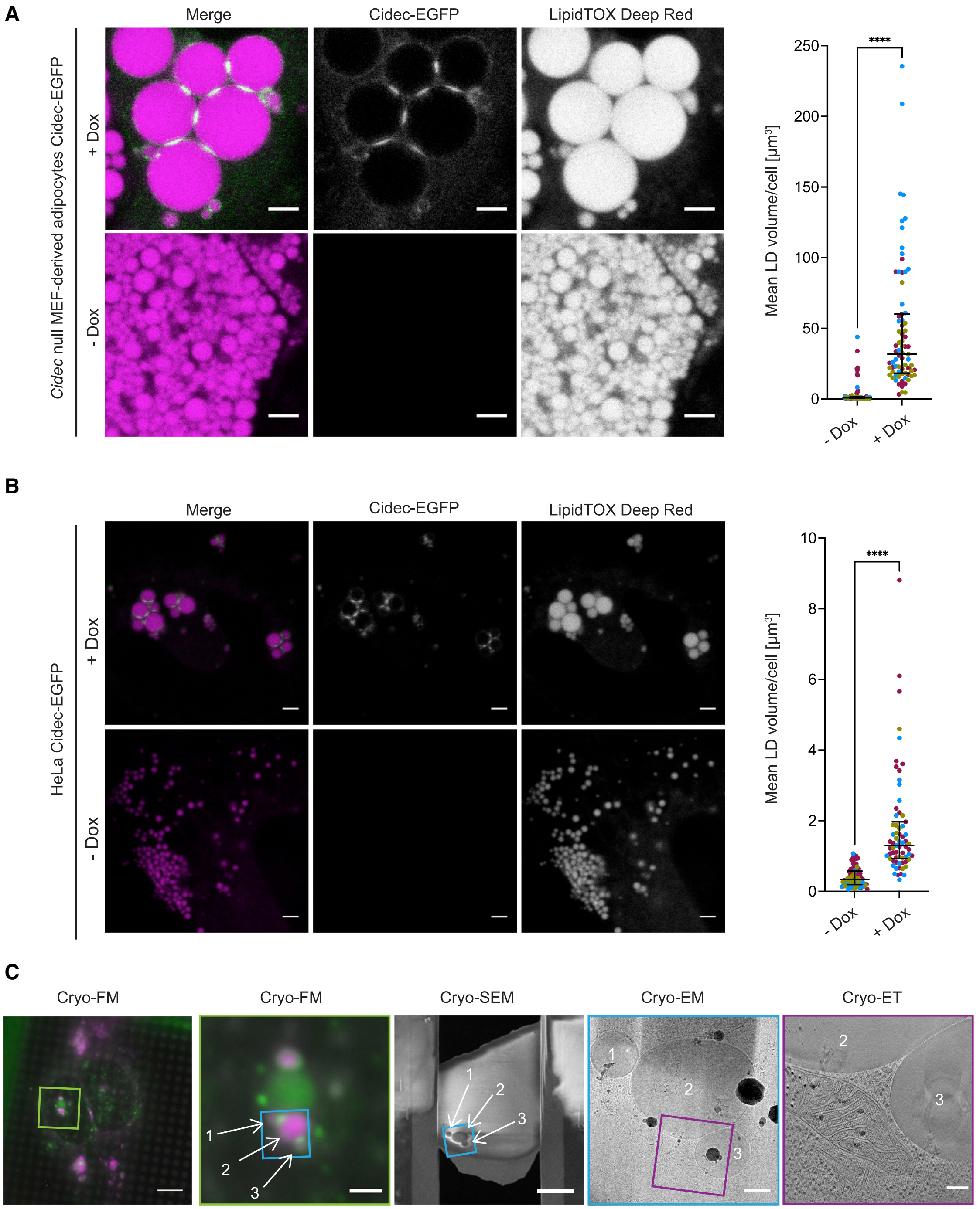

Figure 1. Induced expression of Cidec-EGFP increases LD sizes in *Cidec* null MEF-derived adipocytes and in HeLa cells

(A) FM of fixed *Cidec* null MEF-derived adipocytes inducibly expressing Cidec-EGFP (green). LDs were labeled with LipidTOX Deep Red dye (magenta). *Cidec* null MEFs were differentiated into mature adipocytes over the course of 10 days in the continuous presence (upper) or absence (lower) of doxycycline. The plot shows mean LD volumes per cell. Dots represent individual cells, color coded according to experiment. Black lines correspond to the median of all cells with interquartile range (IQR) (n = 75 per condition, from three experiments: −Dox 0.86 μm^3, IQR 0.98 μm^3; +Dox 31.79 μm^3, IQR 41.81 μm^3).

(B) FM of fixed HeLa cells inducibly expressing Cidec-EGFP (green). LDs were stained with LipidTOX Deep Red dye (magenta). (Upper) HeLa cells induced with doxycycline for Cidec-EGFP expression. (Lower) HeLa cells in the absence of doxycycline induction. Cells were fixed 24 h after the addition of oleic acid and doxycycline. The plot shows mean LD volumes per cell. Dots represent individual cells, color coded according to experiment. Black lines correspond to the median of all cells with IQR (−Dox, n = 69, from three experiments, median 0.34 μm^3, IQR 0.39 μm^3; +Dox, n = 67, from three experiments, median 1.30 μm^3, IQR 1.04 μm^3).

(legend continued on next page)

results show that induced expression of Cidec-EGFP in MEF-derived *Cidec* null adipocytes recapitulates the localization and activity of Cidec on LDs, consistent with previous observations in other cell types.[6,8–10,12] We sought to exploit this experimental paradigm to investigate the architecture of the interface between LDs by cellular electron cryo-tomography (cryo-ET). Previous immunoelectron microscopy showed the accumulation of Cidec between LDs, hence the interfaces were termed contact sites, but it had remained elusive whether the cores of the contacting LDs are continuous or the monolayers are fused.[8] Recent developments in cellular cryo-ET provide unprecedented sample preservation and resolution to visualize cellular interiors in 3D.[13] These methodological advances are ideal to resolve cellular structures such as LD monolayers in an unperturbed, near-native state.[14,15] MEF-derived adipocytes are too "thick" to be directly visualized by cryo-ET and require growth to high confluency, which renders vitrification difficult. Therefore, we transferred the Tet-ON inducible expression of Cidec-EGFP to HeLa cells, for which cryo-focused ion beam (cryo-FIB) milling workflows to thin cells for cryo-ET are well established.[13,16] Expression of Cidec in heterologous cells that do not express any adipocyte-specific proteins induces LD enlargement, indicating that Cidec alone is sufficient for this process.[8–10] We "fed" Cidec-EGFP-expressing HeLa cells with oleic acid for 24 h to trigger LD formation. Although LDs in HeLa cells are considerably smaller than in MEF-derived adipocytes, Cidec-EGFP expression increased LD volumes approximately 4.5-fold (Figure 1B) (median −Dox, 0.34 μm^3, n = 69 cells; median +Dox, 1.3 μm^3, n = 67 cells, $p < 0.0001$), whereas expression of cytosolic EGFP had no effect (Figure S1B). These results verify that expression of Cidec-EGFP in HeLa cells is sufficient to replicate its function in LD enlargement.

We vitrified HeLa cells on electron cryo-microscopy (cryo-EM) grids after feeding them with oleic acid and inducing Cidec-EGFP expression. To identify LD interfaces at which Cidec-EGFP is enriched, we imaged the vitrified cells by fluorescence microscopy (FM) at cryogenic temperatures (cryo-FM) (Figure 1C). Subsequently, we thinned corresponding cell regions by cryo-FIB milling and subjected them to cryo-ET (Figure 1C).[13] This approach ensured that we visualized *bona fide* Cidec-EGFP-marked LD interfaces in a near-native state and at high resolution. In the resulting tomograms, we observed LDs with neutral lipid cores of dense, amorphous appearance (Figure 2), previously attributed to a mixture of triacylglycerol (TAG) and small amounts of cholesterol esters.[14] The surrounding monolayers were resolved as single dark lines, compared with the typical two lines visible for bilayers (Figure S2A). We frequently found mitochondria and endoplasmic reticulum (ER) in close proximity to the LDs, likely representing organelle contact sites important for lipid metabolism (Figure 2).[17] Where two LDs were closely apposed, we often observed deformations of the otherwise nearly spherical shape of the LDs. These large-scale morphologies varied among the interfaces we imaged and corresponded to either minimal deformation (Figure 2A; Video S1), flattening of both LDs (Figure 2B; Video S2), one LD locally protruding and inducing an indentation in the other LD (Figure 2C; Video S3), or the smaller LD locally imposing its curvature by inducing an indentation in the larger LD (Figure 2D; Video S4).

In all cases, the two phospholipid monolayers appeared as separate entities. We determined that the mean distance between closely apposed monolayers forming LD-LD interfaces was 10.9 nm (n = 21 interfaces, SD 2.2 nm) (see STAR Methods and Figure S4B). Furthermore, we often observed a dense layer of material between the monolayers, likely corresponding to proteins (Figures 2A–2D, third panel). Given the dimensions of the interface and the dense protein packing, we hypothesized that the bulkiness of the Cidec-EGFP construct might influence the architecture of the interface.

To address the influence of the EGFP moiety, we generated a HeLa cell line in which we inducibly expressed untagged Cidec. Using cryo-FM, we identified LipidTOX-labeled LDs closely apposed to each other, hence likely engaged in an LD-LD interface (Figures S2B and S2C). We targeted these areas by cryo-FIB milling (Figure S2D) and subsequent cryo-ET. We found that the overall architecture of LD interfaces was consistent between HeLa cells expressing Cidec-EGFP and untagged Cidec (Figures 3A–3C). As for Cidec-EGFP, the two apposed monolayers were visible as two separate entities in cells expressing untagged Cidec (Figures 3A–3C, third panels; Videos S5–S7).

The LD pairs in cells expressing untagged Cidec displayed similar large-scale morphologies to those observed for Cidec-EGFP, indicating that these deformations are inherent to Cidec-mediated LD-LD interfaces (Figures 3A–3C; Videos S5–S7). We thus analyzed whether the different morphologies correlated with the ratio of diameters of the LDs engaged in the interface, or with the absolute sizes of the LDs (see STAR Methods). Minimal deformation, flattening, and protruding LDs were morphologies found at ratios of large-to-small LD diameter between 1 and 2.5 (Figure 3D). In contrast, small undeformed LDs forming an indentation in large LDs were found when the diameter ratio was between 3.6 and 5.7. This type of morphology was exclusively associated with LDs of 400 nm or less in diameter, and such small LDs were never associated with other morphologies. These data suggest that, at diameters of less than 400 nm, LDs maintain a spherical shape when interacting with large LDs and impose their curvature locally on the interacting LD. The variability associated with all other morphologies indicates that, when LDs are larger than 400 nm, deformations do not depend on the sizes

(C) HeLa cells inducibly expressing Cidec-EGFP (green) and LDs labeled with LipidTOX Deep Red (magenta), grown on a cryo-EM grid and imaged by cryo-FM (first and second panel). Second panel corresponds to a magnified view of the area indicated with a green square in the first panel. Regions for cryo-FIB milling were chosen based on LD size (white arrows) and Cidec-EGFP enrichment at LD interfaces. Note that the large, round, green signal likely corresponds to autofluorescence, known to occur in mammalian cells imaged by cryo-FM.[11] Third panel: Cryo-SEM overview image of the lamella generated by cryo-FIB milling from the HeLa cell shown in first and second panel. Interacting LDs identified by cryo-FM are visible in the resulting lamella (white arrows). Milled regions were imaged by cryo-EM to identify LDs in close proximity to each other (fourth panel) and then subjected to cryo-ET (fifth panel, corresponds to the same virtual tomographic slice as shown in Figure 2B, third panel). Corresponding areas in different images are indicated by blue and purple squares, respectively. Scale bars in (A) and (B), 2.5 μm. Scale bars in (C), from left to right, 10 μm, 2.5 μm, 5 μm, 500 nm, 150 nm.

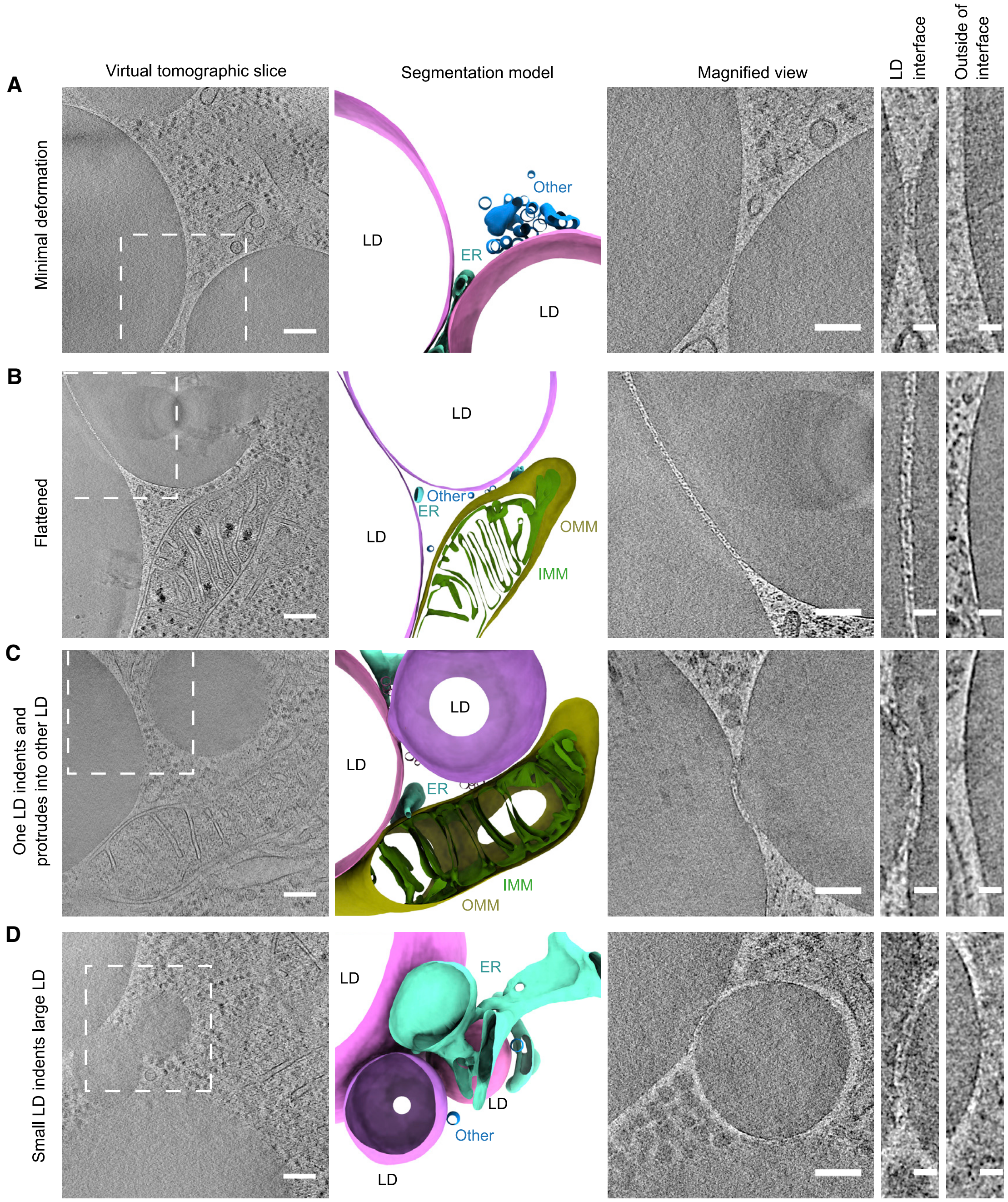

Figure 2. Electron cryo-tomography of LD interfaces in HeLa cells expressing Cidec-EGFP

(A–D) HeLa cells inducibly expressing Cidec-EGFP were vitrified, screened for LD interfaces by cryo-FM, thinned by cryo-FIB milling, and imaged by cryo-ET. The shape of the LDs is deformed where two LDs are closely apposed. The different observed morphologies are classified as follows: (A) minimal deformation of the monolayers; (B) flattening of both LDs forming an interface, same example as shown in Figure 1C; (C) protrusion of one LD into the other LD, resulting in an indentation; (D) indentation of the larger LD caused by the smaller LD. First column from left: virtual slices through tomograms acquired at areas where LDs are in close proximity (Videos S1–S4). Dashed squares indicate areas corresponding to magnified images in third column. Second column: segmentation models of LDs and membranous organelles in proximity to LDs. Pink and magenta shades, LDs; yellow, outer mitochondrial membrane (OMM); green, inner mitochondrial membrane (IMM); turquoise, ER; blue, other membranous organelles (Other). Third column: close-ups of interfaces between LDs. Note that these are different virtual slices than in the first column. Fourth and fifth columns: close-ups of monolayers at the interface (fourth column) and outside the interface (fifth column). In (A), the third, fourth, and fifth panels are derived from the same virtual slice. In (C), the third and fifth panels are derived from the same virtual slice. In (D), the third and fourth panels are derived from the same virtual slice.

Scale bars in (A)–(D), 150 nm in first column, 100 nm in third column, 25 nm in fourth and fifth columns.

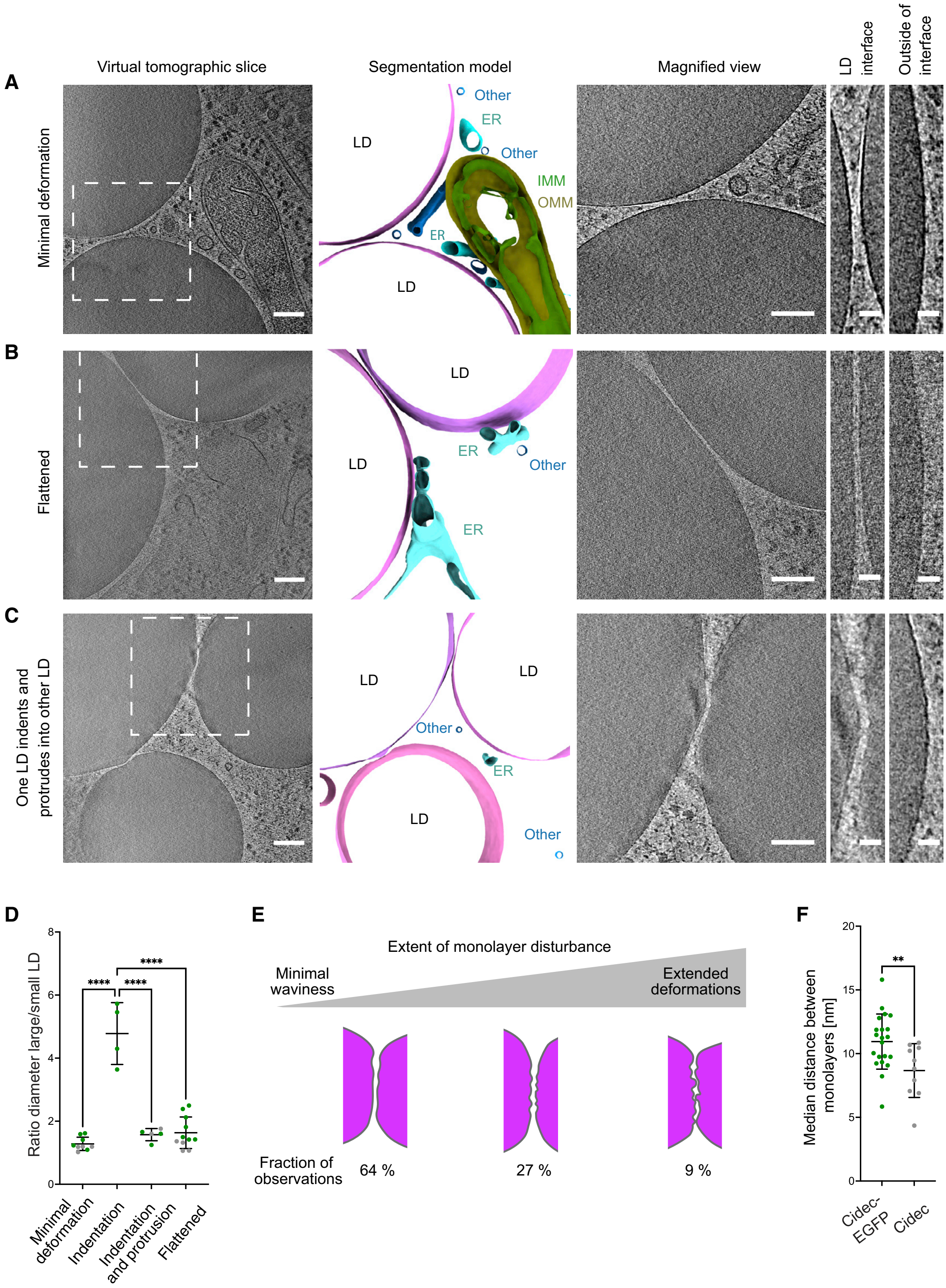

Figure 3. Electron cryo-tomography of LD interfaces in HeLa cells expressing Cidec

(A–C) HeLa cells inducibly expressing untagged Cidec were vitrified, screened for enlarged LDs in close proximity by cryo-FM, thinned by cryo-FIB milling, and imaged by cryo-ET. The shape of the LDs is deformed where two LDs are closely apposed. The different observed morphologies are classified as follows: (A) minimal deformation of the monolayers; (B) flattening of both LDs, forming an interface; (C) protrusion of one LD into the other LD, resulting in an indentation. First column from left: virtual slices through tomograms acquired at areas where LDs are in close proximity (Videos S5–S7). Dashed squares indicate areas

(legend continued on next page)

of interacting LDs (diameter ratio of small LDs indenting large LD versus any other morphology, $p < 0.0001$).

The close apposition of LDs and the appearance of an interface could potentially be caused by crowding of the large amounts of LDs within the cell, rather than by Cidec-mediated tethering. To address this possibility, we acquired cryo-EM data from cells fed with oleic acid in which we did not induce Cidec-EGFP. We found that, in the absence of Cidec-EGFP, there was minimal clustering of LDs (Figures S3A and S3D). In cells expressing Cidec-EGFP (Figures S3A and S3B) or untagged Cidec (Figures S3A and S3C), more than half of the LDs observed in overview images were engaged in an interface, whereas only about 15% of LDs in cells not expressing Cidec-EGFP were in close proximity to each other (Figures S3A and S3D) ($p = 0.0002$). In keeping with the more scattered distribution of LDs in cells not expressing Cidec-EGFP, we rarely detected LDs in overview cryo-EM images of lamellae from these cells, resulting in a small total number of LDs (Figure S3A). These data support previous findings suggesting that Cidec promotes tethering of LDs,[7] and indicate that the occurrence of LD-LD interfaces is a consequence of Cidec expression.

Neither for Cidec-EGFP nor for untagged Cidec did we observe a continuity between the two apposing monolayers that would be indicative of a stable fusion or pore formation. While the apposing monolayers seemed complete, their appearance within the LD interface sometimes differed from the appearance on the rest of the LD surface (Figures 2A–2D and 3A–3C, fourth and fifth panels). In areas not involved in interfaces, the monolayers often appeared smooth, whereas, within the interfaces, the monolayers exhibited waviness and nanometer-scale irregularities, indicating local disturbances. The extent of the disturbances varied between different interfaces (Figure 3E) and was only assessed in tomograms with optimally vitrified interfaces (see STAR Methods). While the majority of assessed interfaces showed rather minimal waviness (Figures 2A and 3A), 9% displayed more extensive deformations of the monolayer (Figures 2C and 2D) ($n = 22$ interfaces). Within the interfaces with extended deformations, the disturbances were not evenly distributed over the entire monolayer. Instead, these interfaces exhibited areas with variedly pronounced deformations (Figure S4A). Furthermore, the distances we measured between the monolayers varied throughout the contact area, also indicating local heterogeneity within the interfaces (Figures S4B–S4F). These observations suggest that TAG transfer is likely to occur through two locally disturbed, yet largely intact, phospholipid monolayers.

When we measured the distances between the monolayers, we found that, overall, the monolayers were closer for untagged Cidec than for Cidec-EGFP (mean of distances: untagged Cidec 8.7 nm, SD 2.1 nm, $n = 10$ interfaces, $p = 0.010$ compared with Cidec-EGFP) (Figure 3F). The observed difference compared with Cidec-EGFP interfaces is in good agreement with the dimensions of EGFP molecules,[18] suggesting that the increase in distance is due to the space taken up by EGFP moieties. Furthermore, the layer between the monolayers displayed lower visibility and density than for Cidec-EGFP, possibly in part reflecting the smaller molecular weight of the construct. These results suggest that the spacing between LD monolayers is determined by dense packing of Cidec molecules and is influenced by the size of the Cidec construct mediating the interaction.

Having identified determinants of the architecture of the LD interface, we next sought to link them to neutral lipid transfer function. We hypothesized that, if Cidec-EGFP has an effect due to the bulky size of the tag (27 kDa) compared with untagged Cidec, Cidec conjugated to SUMOstar may have a reduced effect due to the intermediate size of the tag (12 kDa). By titrating doxycycline dosage, comparable *Cidec*, *Cidec-SUMOstar*, and *Cidec-EGFP* transcript levels were confirmed for all three stable HeLa cell lines (Figure S5A). For FM imaging, we fixed cells expressing untagged Cidec and Cidec-SUMOstar 24 h after oleic acid and doxycycline were added (Figure 4A). Similarly to Cidec-EGFP (Figure 1B), inducing expression of either Cidec-SUMOstar or Cidec increased the LD volume ($p < 0.0001$ for Cidec-SUMOstar, $p < 0.0001$ for Cidec; Figure 4B). All three constructs reduced the number of LDs per cell ($p < 0.0001$ for Cidec-EGFP, $p < 0.0001$ for Cidec-SUMOstar, $p < 0.0001$ for Cidec; Figure 4C). No increase in LD volume ($p = 0.9933$, Figure 4B) or reduction in LD number ($p = 0.8477$, Figure 4C) was observed upon induction of cytosolic EGFP expression. When comparing cells expressing the different constructs, we found that expression of untagged Cidec resulted in the greatest increase in LD volume (median of mean LD volumes/cell: EGFP, 0.31 μm^3; Cidec-EGFP, 1.30 μm^3; Cidec-SUMOstar, 2.05 μm^3; Cidec, 3.30 μm^3; $p < 0.0001$ for group comparison; n : 67–73 cells) (Figure 4B) and strongest reduction in LD number (median of mean LD number/cell: EGFP 108 LDs/cell, Cidec-EGFP 45 LDs/cell, Cidec-SUMOstar 28 LDs/cell, Cidec 18 LDs/cell; $p < 0.0001$

corresponding to magnified images in third column. Second column: segmentation models of LDs and membranous organelles in proximity of LDs. Pink and magenta shades, LDs; yellow, OMM; green, IMM; turquoise, ER; blue, other membranous organelles (Other). Third column: close-ups of interfaces between LDs (different virtual slices than in the first column). Fourth and fifth column: close-ups of monolayers at the interface (fourth column; in A and C this is the same virtual slice as in the third column) and outside the interface (fifth column).

(D) The different interface morphologies plotted as a ratio of the diameter of the larger LD to the diameter of the smaller LD. Green dots indicate Cidec-EGFP data, gray dots indicate untagged Cidec data. Black lines represent mean with SD (minimal deformation, $n = 9$, mean 1.28, SD 0.21; indentation, $n = 4$, mean 4.78, SD 0.98; indentation and protrusion, $n = 5$, mean 1.57, SD 0.19; flattened, $n = 11$, mean 1.63, SD 0.50). Only LD pairs where a reliable diameter measurement was possible (see STAR Methods) were included in this quantification. Two interfaces, both from the same Cidec-EGFP tomogram, were therefore excluded.

(E) Model representation of the observed monolayer disturbances at LD interfaces from both Cidec-EGFP and untagged Cidec data combined, binned into three arbitrary classes to group minimal, intermediate and maximal deformations. Only LD-LD interfaces in which the monolayers were clearly visible and well preserved both within and outside the interface were considered in this analysis ($n = 22$ interfaces). Four interfaces from two Cidec-EGFP tomograms and five interfaces from three untagged Cidec tomograms were excluded due to insufficient vitrification. Among those excluded is the tomogram shown in (C).

(F) Median distances between each LD pair, for Cidec-EGFP (green dots) and untagged Cidec (gray dots). Black lines represent the mean with SD (Cidec-EGFP, 10.9 nm, SD 2.2 nm, $n = 21$ interfaces; Cidec, 8.7 nm, SD 2.1 nm, $n = 10$ interfaces).

Scale bars in (A)–(C), 150 nm in first column, 100 nm in third column, 25 nm in fourth and fifth columns.

CellPress
OPEN ACCESS

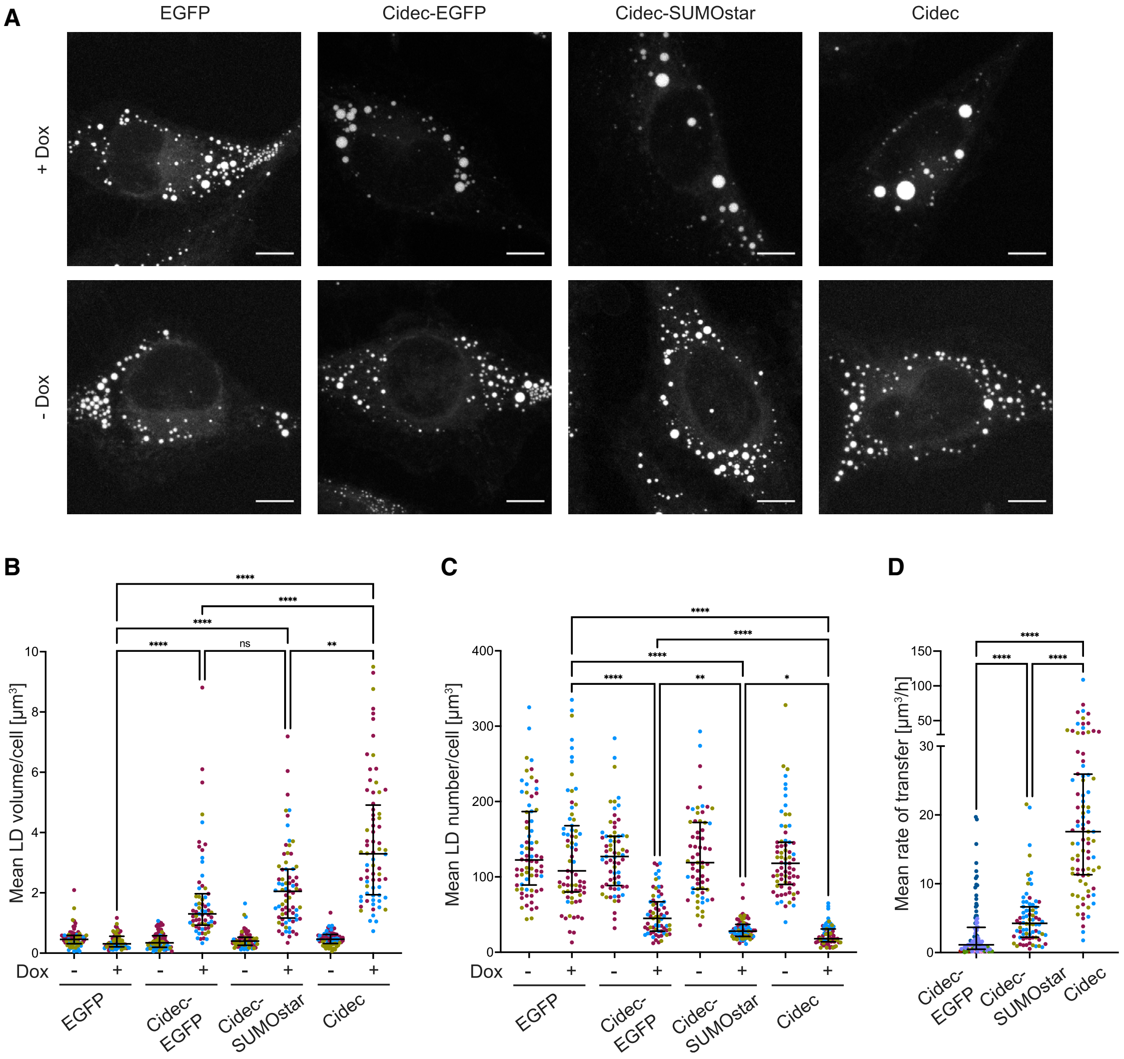

Figure 4. Quantitative FM of HeLa cells expressing Cidec-EGFP, Cidec-SUMOstar, or untagged Cidec

(A) Representative FM images of fixed HeLa cells, loaded with oleic acid, either doxycycline induced (+Dox) or uninduced (−Dox) for EGFP, Cidec-EGFP, Cidec-SUMOstar, or Cidec expression for 24 h prior to fixing and imaging. Scale bars, 10 μm.

(B and C) Quantification of mean LD volume per cell (B) or mean LD number per cell (C) from 3D FM images of fixed HeLa cells, loaded with oleic acid, either uninduced (−Dox) or doxycycline induced (+Dox) for EGFP, Cidec-EGFP, Cidec-SUMOstar, or Cidec expression 24 h prior to fixing and imaging. Each dot represents the mean LD volume (B) or LD number (C) in one cell. Different colors correspond to three different experimental repeats. In each experiment, 11–35 cells were analyzed per condition. Black lines represent the medians of all data points with IQR. Medians in (B), EGFP, −Dox 0.46 μm^3, IQR 0.28 μm^3; +Dox 0.31 μm^3, IQR 0.35 μm^3; Cidec-EGFP, −Dox 0.34 μm^3, IQR 0.39 μm^3; +Dox 1.30 μm^3, IQR 1.04 μm^3; Cidec-SUMOstar, −Dox 0.40 μm^3, IQR 0.27 μm^3 +Dox 2.05 μm^3, IQR 1.62 μm^3; Cidec, −Dox 0.46 μm^3, IQR 0.3 μm^3, +Dox 3.30 μm^3, IQR 2.98 μm^3. Cidec-EGFP data are the same as plotted in Figure 1B. Medians in (C), EGFP, −Dox 122.5 LDs/cell, IQR 97.55 LDs/cell, +Dox 108 LDs/cell, IQR 88.0 LDs/cell; Cidec-EGFP, −Dox 127.0 LDs/cell, IQR 65.5 LDs/cell, +Dox 45.0 LDs/cell, IQR 39 LDs/cell; Cidec-SUMOstar, −Dox 119.0 LDs/cell, IQR 88.6 LDs/cell, +Dox 28.0 LDs/cell, IQR 16 LDs/cell, Cidec, −Dox 118.0 LDs/cell, IQR 56 LDs/cell, +Dox 18.0 LDs/cell, IQR 17 LDs/cell.

(D) The rate of lipid transfer from donors to acceptors, as the average change of donor volume over the course of the transfer event. Measurements were done three-dimensionally in live cells imaged by time-lapse FM of LipidTOX signals (Videos S8–S10). Live FM started 2 h after doxycycline-induced Cidec and Cidec-SUMOstar expression, and 24 h after doxycycline-induced Cidec-EGFP expression. Lipid transfer rates of individual events are represented by dots, color coded according to experimental repeat. Black lines represent median with IQR (Cidec-EGFP, 1.12 μm^3/h, IQR 3.18 μm^3/h, n = 104 from five independent experiments each analyzing 4–43 cells; Cidec-SUMOstar, 4.25 μm^3/h, IQR 4.42 μm^3/h, n = 76 from three independent experiments each analyzing 18–36 cells; Cidec, mean 17.57 μm^3/h, IQR 14.62 μm^3/h, n = 88 from three independent experiments each analyzing 23–34 cells). Only donors involved in active lipid transport and in contact with a single acceptor were considered.

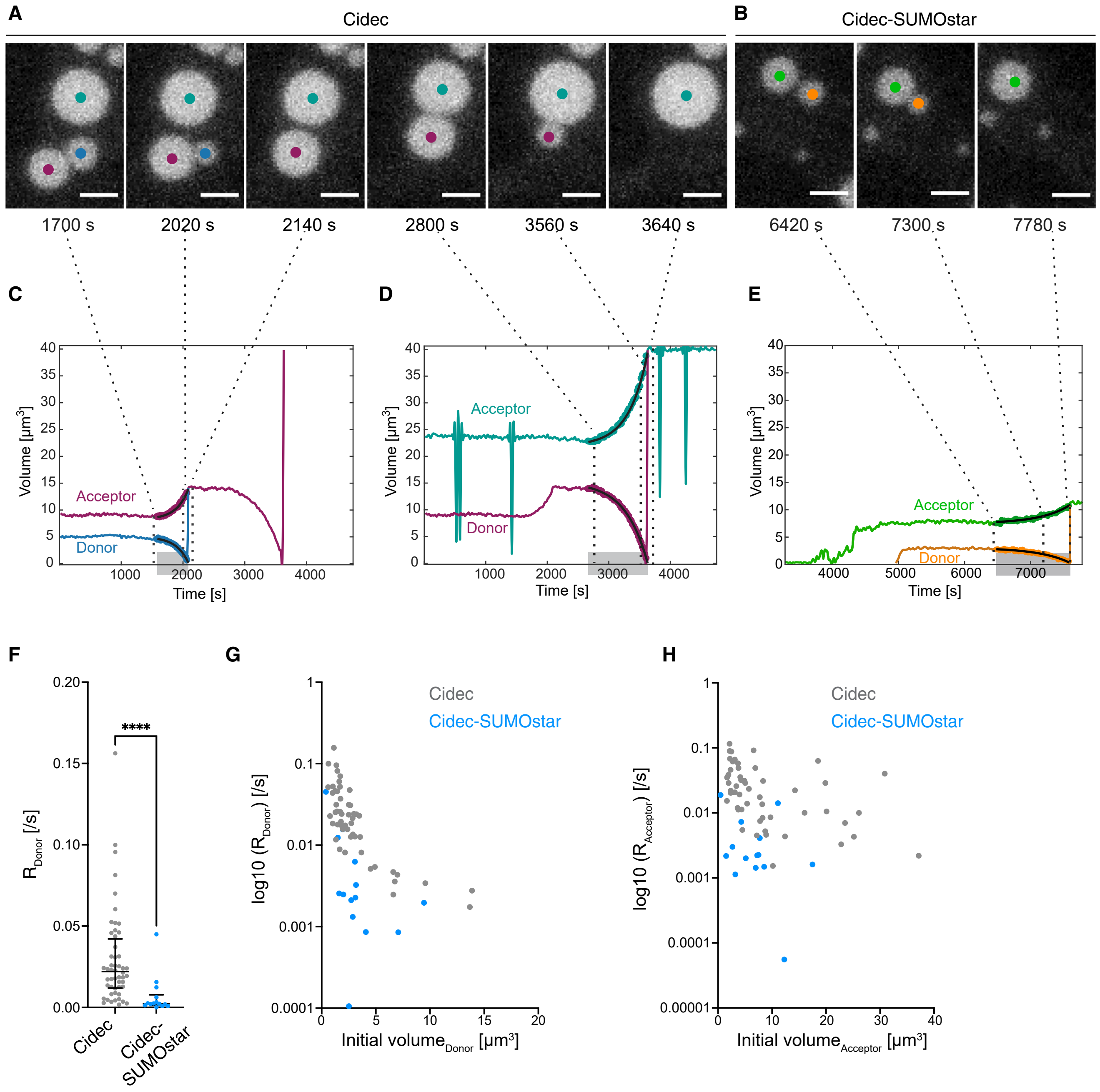

Figure 5. Analysis of LD volume changes over time shows that neutral lipid transfer follows exponential kinetics

(A and B) Time course live FM of HeLa cells expressing untagged Cidec (A) and Cidec-SUMOstar (B). Representative LD pairs engaged in active lipid transfer. Panels are maximum projections of a z-stack. Scale bars, 2.5 µm.

(C–E) Individual LDs were segmented out and tracked over the course of the movies. Traces indicate the volume change of individual LDs over time, in Cidec- (C and D) and Cidec-SUMOstar-expressing (E) cells. Gray overlays on x axes highlight the time when LDs are in contact ("event time") and involved in active transfer. Overlaid on the traces, dots represent the individual time points when the two LDs were detected as being in close contact (both LDs detected within the same pixel). Black lines overlaid on the dots correspond to the exponential fit, which is used to determine R values (shown in F).

(F) The R values calculated from the exponential fits of donors for Cidec (n = 52) and Cidec-SUMOstar (n = 14). Each data point corresponds to one transfer event. Black lines correspond to median and IQR (Cidec, n = 52; median, 0.022/s, IQR 0.031; Cidec-SUMOstar, n = 14; median, 0.002/s, IQR 0.007, $p < 0.0001$).

(G) The R values calculated from the exponential fits of donors (as shown in C–E) plotted against the volumes of the donors at the beginning of the transfer event. Each point corresponds to an individual donor. Gray points indicate untagged Cidec and blue points Cidec-SUMOstar.

for group comparison; n : 67–73 cells) (Figure 4C). These results confirm that all three constructs enlarge LDs and reduce their number per cell, albeit with varying efficiency.

We next considered the impact of the constructs on the rate of lipid transfer as this has implications for the *in vivo* physiology of LD growth. Prior work reported that, in 3T3-L1 pre-adipocytes overexpressing Cidec-EGFP, lipid exchange occurred at average rates of 0.13 $\mu m^3/s$, whereas, in differentiated adipocytes expressing endogenous Cidec, average lipid exchange rates of 5.6 $\mu m^3/s$ were reported.[8] These rates described bidirectional lipid exchange. When examining net lipid transfer from the smaller to the larger LD, a rate of 4.8 $\mu m^3/h$ was determined in pre-adipocytes.[8] Thus, assuming a constant net transfer rate, an LD of 5 μm in diameter would require about 14 h to transfer its entire contents to another LD. We collected live-cell FM images of LDs, starting 2 h after induction of expression of Cidec and Cidec-SUMOstar. For Cidec-EGFP, LDs were very small at this time point, so we started live FM 24 h after induction. We identified LD pairs engaged in active lipid transfer by the close proximity of LD cores labeled with LipidTOX (Videos S8–S10). We initially analyzed 100 pairs of juxtaposed LDs in Cidec and Cidec-EGFP-expressing, lipid-loaded cells. The data indicated that >90% of LDs were engaged in active lipid transfer in this context; the implication being that the majority of juxtaposed LDs we have imaged by cryo-ET (Figures 2 and 3) represent active lipid transfer events. All three Cidec constructs mediated lipid transfer in a net directional manner from the smaller LDs (donors) to the larger LDs (acceptors), as expected from previous reports.[7,8] We measured the change of donor volume over time (see STAR Methods). Importantly, only donors that were in contact with a single acceptor were taken into account. For Cidec-EGFP, we found the volume reduction of the donor, and hence the rate of net lipid transfer averaged over the course of the transfer event, to be 1.12 $\mu m^3/h$ (median, n = 104 transfer events) (Figure 4D), again indicating that a complete transfer of neutral lipid content from donors to acceptors required hours, similar to previous reports.[8] For Cidec-SUMOstar, the donor volume reduction rate averaged over the course of the transfer was 4.25 $\mu m^3/h$ (median, n = 76 transfer events), while, in the presence of untagged Cidec, the donor volume reduction rate was 17.57 $\mu m^3/h$ (median, n = 88 transfer events) (Figure 4D). This latter rate is approximately 15 times faster than for Cidec-EGFP ($p < 0.0001$) and indicates that untagged Cidec transfers neutral lipids between donor and acceptor LDs within minutes. For all three constructs, the volume reduction rate averaged over the course of the transfer was higher the larger the initial size of the donor LD (Figures S5B–S5D), consistent with previous findings.[19] Taken together, these data suggest that the average rate of net lipid transfer is considerably slower in cells expressing Cidec with a bulkier tag and greater distance between the monolayers, although it is possible that properties in addition to simple size of the tag may also be involved.

We next investigated the Cidec-mediated change in LD volume over time by analyzing the kinetics of the process to obtain further insights into the underlying transfer mechanism. Based on the directionality of the net TAG flux from the small to the large LD, it was previously suggested that the process could be pressure driven.[8] We implemented a semi-automated image analysis pipeline (see STAR Methods), which we applied to the live FM data obtained for untagged Cidec and Cidec-SUMOstar. Due to the relatively slow merging of LDs in Cidec-EGFP cells and the resulting use of different imaging settings, we did not include Cidec-EGFP in this analysis. Our analysis automatically determined the time points when a pair of LDs engaging in active lipid transfer first came into contact and when the lipid transfer event was completed (Figures 5A and 5B). For pairs identified as engaged in transfer, we obtained LD volumes in each movie frame and plotted them against time (Figures 5C–5E). The resulting curves showed that the change in LD volume of both donors and acceptors accelerated over time and hence followed exponential kinetics. This was the case both for untagged Cidec (Figures 5C and 5D) and Cidec-SUMOstar (Figure 5E). We fitted an exponential function to the curves and calculated rate constants from the fit (R values), which are a measure of the acceleration of lipid transfer (see STAR Methods). The smaller the R value, the slower the whole transfer event remained over the course of time. Within an LD pair engaged in transfer, the R value of the donor should correspond to the R value of the acceptor, unless the LDs are engaged in more than one transfer event. Hence, for each event, we plotted logarithmically the R values of donor versus acceptor (Figure S5E). For the majority of events, donor and acceptor R values were very similar. To exclude events likely involved in multiple transfers, we further considered only those transfer events for which the absolute value of $\log10(R_{Acceptor})/\log10(R_{Donor}) - 1$ was smaller than 0.4 (Figure S5E, filled circles). Among these events, the median R_{Donor} value was 0.022/s for untagged Cidec and 0.002/s for Cidec-SUMOstar (Cidec, n = 52; Cidec-SUMOstar, n = 14; Figure 5F). Thus collectively, untagged Cidec events showed a faster change of volume over time than Cidec-SUMOstar events ($p < 0.0001$). However, there was considerable variability in R_{Donor} between events. We found that R_{Donor} depended in an exponential fashion on the starting volume of the donor LD (Figure 5G). This effect was not obvious for the starting volume of the acceptor and $R_{Acceptor}$ (Figure 5H). Furthermore, R_{Donor} did not correlate with the ratio between the starting volumes of the acceptor and the donor (Figure S5F). Hence the initial size of the donor LD influences how rapidly the lipid transfer speed increases during a transfer event, while neither the initial size of the acceptor nor the size difference between the two LDs have a dominant influence on the changes in transfer speed.

Furthermore, these results suggest that the transfer of lipids at LD-LD interfaces follows exponential kinetics, starting slowly and accelerating toward the end. While this behavior is robust and retained in the presence of protein tags, the magnitude of the transfer rate is altered by various properties of the protein at the interface. These results are in line with the cryo-ET data, which show that monolayer integrity and overall architecture are consistent

(H) The R values calculated from the exponential fits of acceptors (as shown in C–E) plotted against the volumes of the acceptors at the beginning of the transfer event. Each point corresponds to an individual acceptor. Gray points indicate untagged Cidec and blue points Cidec-SUMOstar. Note that the analysis in this figure is derived from same live-cell imaging data as analyzed in Figure 4D.

features of LD interfaces, while the magnitude of the gap between the monolayers is influenced by the bulkiness of the protein at the interface. The function and organization of Cidec could be further affected by other properties of the EGFP and SUMOstar tags.

The variability in transfer speed and acceleration between individual events is dependent on the volume of the donor at the start of the transfer event. Other contributing factors could be interface morphology and surface area, or the extent of Cidec enrichment at the interface.[19]

DISCUSSION

The contents of LD cores are among the most hydrophobic molecules found in cells. Thus how merging of LDs in the aqueous cytosol is controlled poses a unique cell biological problem. By the close apposition of monolayers, LD interfaces show resemblance to membrane contact sites.[8] However, the distances between monolayers that we report here are considerably smaller than those reported for membrane contact sites involved in lipid transfer.[20] Furthermore, in contrast to lipid transfer proteins at membrane contact sites,[21] Cidec is not known to have a lipid transfer domain. Hence currently available data suggest that the transfer of TAGs between LDs is likely driven by a fundamentally different mechanism from other lipid transfer events. Our observations suggest that transfer of neutral lipids likely occurs through two largely intact monolayers, rather than a stable fusion pore, which would be expected to result in a visible continuity between the monolayers and a mixing of the TAG cores throughout the interface. We cannot entirely exclude the possibility of transient monolayer fusion events on a nanometer scale. However, as transfer occurs within minutes in cells expressing untagged Cidec, and as we have visualized a total of 31 interfaces (including interfaces mediated by both Cidec-EGFP and untagged Cidec) that were likely involved in active transfer, without ever detecting continuity between the two interfacing monolayers, fusion events are rather unlikely in our view. This suggestion is also supported by data reported by Gong et al.[8] showing that, although Cidec is highly mobile on the surface of LDs, it very rarely relocates to a second LD.

Our cryo-ET observations point to the occurrence of molecular-level disturbances in the monolayers, which could reflect local leakiness allowing passage of TAG molecules, in line with previous molecular dynamics simulations.[9] We speculate that the leakiness could be caused by the dense yet uneven packing of Cidec within and between the two monolayers. In accordance with this idea, it has recently been suggested that Cidec accumulates in patchy, lipid-permeable plates between LDs.[19] The overall intactness of the monolayers might be important for retaining a difference in surface tension between the two LDs. This difference generates Laplace pressure, which has been proposed to be the driving force for the transfer.[8,22] Nevertheless, key data confirming such a model were missing. In fact, it has been reported that the lipid transfer rate decreases over time and as the donor volume decreases, suggesting that pressure cannot be the sole driving force.[19,22] Our finding that the transfer accelerates by exponential kinetics provides prerequisite evidence for a pressure-driven transfer model. Moreover, as the pressure is expected to inversely correlate with LD size, our observation that the smaller the donor LD is at the start of the event, the more the transfer accelerates, supports a pressure-driven model. In line with this, our observation that LDs smaller than 400 nm do not deform in shape but impose their curvature on larger LDs suggests that small LDs have a greater internal pressure or surface tension than large LDs. The discrepancy to previous findings may derive from differences in the experimental setup or in the measurement method.[19,22]

In summary, high-spatial-resolution cryo-ET of Cidec-mediated LD interfaces revealed a unique membrane contact site architecture characterized by closely opposed (approximately 10 nm), locally disturbed, but overall intact phospholipid monolayers. Furthermore, high-temporal-resolution live FM revealed that net lipid transfer follows exponential kinetics. These observations are consistent with the transfer of TAGs through the LD monolayers being driven by pressure, and with a form of facilitated Ostwald ripening possibly being the molecular mechanism underpinning the unilocularity of white adipocytes.

Limitations of the study

Here, we have studied the architecture of interfaces between LDs in human cells. As a model system we used HeLa cells instead of differentiated adipocytes. This was necessary since adipocytes require higher cell confluence and feature very large LDs, preventing efficient vitrification and subsequent processing for cryo-ET. The study of overexpressed Cidec in HeLa cells mimics the process of LD coalescence only to a certain extent, as unilocularity is never achieved, and the total amount of neutral lipids stored in LDs per cell is lower than, e.g., in MEF-derived adipocytes. In order to image regions with enlarged LDs, the cells were subjected to thinning by cryo-FIB milling. While cryo-FIB milling is a powerful method to visualize areas deep inside cells by cryo-ET, it is limited in throughput and hence dataset sizes, limiting statistical power. A further drawback of our cryo-ET data is that, for cells expressing untagged Cidec, the presence of the protein at the interfaces could not be confirmed by cryo-FM, rendering data collection less efficient. When collecting the cryo-ET data, presence of LD-LD interfaces was prioritized over ideal sample preservation. Large LDs are located in thick cell areas, which are more difficult to vitrify. Thus, some of our tomograms contained areas of insufficient vitrification and were therefore excluded from certain analyses that required optimal preservation of the monolayers. Furthermore, an intrinsic limitation of cryo-ET is the anisotropic resolution of tomograms. Together with the small size of Cidec (27 kDa), this issue precluded analysis of Cidec structure and organization within the dense protein layer in the LD interfaces. Another limitation of this study is that we could not determine the protein expression levels for the different Cidec constructs due to the lack of suitable antibodies. However, the mRNA levels of all three used constructs are similar, and we do not expect differences in protein levels to affect our conclusions.

STAR★METHODS

Detailed methods are provided in the online version of this paper and include the following:

- KEY RESOURCES TABLE

- RESOURCE AVAILABILITY
  - Lead contact
  - Materials availability
  - Data and code availability
- EXPERIMENTAL MODEL AND SUBJECT DETAILS
  - Cell lines
- METHOD DETAILS
  - Cloning
  - Generation of *Cidec* null MEF and HeLa cell lines with Doxycycline-inducible expression of Cidec constructs
  - Determination of mRNA transcript levels of *Cidec* constructs in HeLa cells
  - Fixed cell imaging
  - Analysis of LD sizes and number in fixed cell fluorescence images
  - Live cell imaging
  - Quantification of frequency of active lipid transfer events
  - Manual analysis of lipid transfer rate
  - Semi-automated analysis of lipid transfer rate
  - Sample preparation for cryo-FM and cryo-FIB milling
  - Cryo-FIB milling
  - Electron cryo-tomography (cryo-ET)
  - Analysis of distances between lipid droplets
  - Determination of lipid droplet diameters
  - Determination of degree of monolayer deformation
- QUANTIFICATION AND STATISTICAL ANALYSIS

SUPPLEMENTAL INFORMATION

Supplemental information can be found online at https://doi.org/10.1016/j.celrep.2023.112107.

ACKNOWLEDGMENTS

We would like to thank the MRC LMB electron microscopy facility for support in cryo-FIB milling and cryo-ET data acquisition. We thank Christel Genoud for access to cryo-FIB milling at the University of Lausanne. We thank Piotr Szwedziak for helpful discussions. Work in the group of W.K. was supported by the Medical Research Council, as part of United Kingdom Research and Innovation (also known as UK Research and Innovation) under award MC_UP_1201/8; by the University of Bern; the NCCR TransCure, a National Center of Competence in Research of the Swiss National Science Foundation (SNSF) (185544); and the SNSF (project 201158). For the purpose of open access, the authors have applied a CC BY public copyright license to any author accepted manuscript version arising. D.B.S. is supported by the Wellcome Trust (WT 219417), the MRC Metabolic Disease Unit (MRC_MC_UU_12012.1), and The National Institute for Health Research (NIHR) Cambridge Biomedical Research Centre and NIHR Rare Disease Translational Research Collaboration. The IMS MRL Imaging Core is supported by a Wellcome Trust Major Award (208363/Z/17/Z). We acknowledge the access and services provided by the EMBL Structural and Computational Biology Unit and by the EMBL Imaging Center, supported by the Boehringer Ingelheim Foundation.

AUTHOR CONTRIBUTIONS

K.L. and I.G., conceptualization, methodology, investigation, formal analysis, and writing – original draft; J.B., custom software development, formal analysis, and writing – review & editing; P.C.H. and W.J.H.H., data acquisition and writing – review & editing; O.M. and A.C.B., sample preparation and writing – review & editing; W.K. and D.B.S., conceptualization, methodology, investigation, formal analysis, supervision, and writing – original draft.

DECLARATION OF INTERESTS

The authors declare no competing interests.

Received: August 9, 2021
Revised: October 14, 2022
Accepted: January 30, 2023
Published: February 16, 2023

REFERENCES

1. Walther, T.C., and Farese, R.V., Jr. (2012). Lipid droplets and cellular lipid metabolism. Annu. Rev. Biochem. *81*, 687–714. https://doi.org/10.1146/annurev-biochem-061009-102430.
2. Nishino, N., Tamori, Y., Tateya, S., Kawaguchi, T., Shibakusa, T., Mizunoya, W., Inoue, K., Kitazawa, R., Kitazawa, S., Matsuki, Y., et al. (2008). FSP27 contributes to efficient energy storage in murine white adipocytes by promoting the formation of unilocular lipid droplets. J. Clin. Invest. *118*, 2808–2821. https://doi.org/10.1172/JCI34090.
3. Toh, S.Y., Gong, J., Du, G., Li, J.Z., Yang, S., Ye, J., Yao, H., Zhang, Y., Xue, B., Li, Q., et al. (2008). Up-regulation of mitochondrial activity and acquirement of brown adipose tissue-like property in the white adipose tissue of fsp27 deficient mice. PLoS One *3*, e2890. https://doi.org/10.1371/journal.pone.0002890.
4. Rubio-Cabezas, O., Puri, V., Murano, I., Saudek, V., Semple, R.K., Dash, S., Hyden, C.S.S., Bottomley, W., Vigouroux, C., Magré, J., et al. (2009). Partial lipodystrophy and insulin resistant diabetes in a patient with a homozygous nonsense mutation in CIDEC. EMBO Mol. Med. *1*, 280–287. https://doi.org/10.1002/emmm.200900037.
5. Zhou, L., Park, S.Y., Xu, L., Xia, X., Ye, J., Su, L., Jeong, K.H., Hur, J.H., Oh, H., Tamori, Y., et al. (2015). Insulin resistance and white adipose tissue inflammation are uncoupled in energetically challenged Fsp27-deficient mice. Nat. Commun. *6*, 5949. https://doi.org/10.1038/ncomms6949.
6. Puri, V., Konda, S., Ranjit, S., Aouadi, M., Chawla, A., Chouinard, M., Chakladar, A., and Czech, M.P. (2007). Fat-specific protein 27, a novel lipid droplet protein that enhances triglyceride storage. J. Biol. Chem. *282*, 34213–34218. https://doi.org/10.1074/jbc.M707404200.
7. Jambunathan, S., Yin, J., Khan, W., Tamori, Y., and Puri, V. (2011). FSP27 promotes lipid droplet clustering and then fusion to regulate triglyceride accumulation. PLoS One *6*, e28614. https://doi.org/10.1371/journal.pone.0028614.
8. Gong, J., Sun, Z., Wu, L., Xu, W., Schieber, N., Xu, D., Shui, G., Yang, H., Parton, R.G., and Li, P. (2011). Fsp27 promotes lipid droplet growth by lipid exchange and transfer at lipid droplet contact sites. J. Cell Biol. *195*, 953–963. https://doi.org/10.1083/jcb.201104142.
9. Barneda, D., Planas-Iglesias, J., Gaspar, M.L., Mohammadyani, D., Prasannan, S., Dormann, D., Han, G.S., Jesch, S.A., Carman, G.M., Kagan, V., et al. (2015). The brown adipocyte protein CIDEA promotes lipid droplet fusion via a phosphatidic acid-binding amphipathic helix. Elife *4*, e07485. https://doi.org/10.7554/eLife.07485.
10. Price, A.M., Doner, N.M., Gidda, S.K., Jambunathan, S., James, C.N., Schami, A., Yurchenko, O., Mullen, R.T., Dyer, J.M., Puri, V., and Chapman, K.D. (2020). Mouse Fat-Specific Protein 27 (FSP27) expressed in plant cells localizes to lipid droplets and promotes lipid droplet accumulation and fusion. Biochimie *169*, 41–53. https://doi.org/10.1016/j.biochi.2019.08.002.
11. Carter, S.D., Mageswaran, S.K., Farino, Z.J., Mamede, J.I., Oikonomou, C.M., Hope, T.J., Freyberg, Z., and Jensen, G.J. (2018). Distinguishing signal from autofluorescence in cryogenic correlated light and electron microscopy of mammalian cells. J. Struct. Biol. *201*, 15–25. https://doi.org/10.1016/j.jsb.2017.10.009.
12. Sun, Z., Gong, J., Wu, H., Xu, W., Wu, L., Xu, D., Gao, J., Wu, J.W., Yang, H., Yang, M., and Li, P. (2013). Perilipin1 promotes unilocular lipid droplet

formation through the activation of Fsp27 in adipocytes. Nat. Commun. *4*, 1594. https://doi.org/10.1038/ncomms2581.

13. Mahamid, J., Pfeffer, S., Schaffer, M., Villa, E., Danev, R., Cuellar, L.K., Förster, F., Hyman, A.A., Plitzko, J.M., and Baumeister, W. (2016). Visualizing the molecular sociology at the HeLa cell nuclear periphery. Science *351*, 969–972. https://doi.org/10.1126/science.aad8857.
14. Mahamid, J., Tegunov, D., Maiser, A., Arnold, J., Leonhardt, H., Plitzko, J.M., and Baumeister, W. (2019). Liquid-crystalline phase transitions in lipid droplets are related to cellular states and specific organelle association. Proc. Natl. Acad. Sci. USA *116*, 16866–16871. https://doi.org/10.1073/pnas.1903642116.
15. Rogers, S., Gui, L., Kovalenko, A., Zoni, V., Carpentier, M., Ramji, K., Ben Mbarek, K., Bacle, A., Fuchs, P., Campomanes, P., et al. (2022). Triglyceride lipolysis triggers liquid crystalline phases in lipid droplets and alters the LD proteome. J. Cell Biol. *221*, e202205053. https://doi.org/10.1083/jcb.202205053.
16. Ader, N.R., Hoffmann, P.C., Ganeva, I., Borgeaud, A.C., Wang, C., Youle, R.J., and Kukulski, W. (2019). Molecular and topological reorganizations in mitochondrial architecture interplay during Bax-mediated steps of apoptosis. Elife *8*, e40712. https://doi.org/10.7554/eLife.40712.
17. Schuldiner, M., and Bohnert, M. (2017). A different kind of love - lipid droplet contact sites. Biochim. Biophys. Acta. Mol. Cell Biol. Lipids *1862*, 1188–1196. https://doi.org/10.1016/j.bbalip.2017.06.005.
18. Yang, F., Moss, L.G., and Phillips, G.N., Jr. (1996). The molecular structure of green fluorescent protein. Nat. Biotechnol. *14*, 1246–1251. https://doi.org/10.1038/nbt1096-1246.
19. Lyu, X., Wang, J., Wang, J., Yin, Y.S., Zhu, Y., Li, L.L., Huang, S., Peng, S., Xue, B., Liao, R., et al. (2021). A gel-like condensation of Cidec generates lipid-permeable plates for lipid droplet fusion. Dev. Cell *56*, 2592–2606.e7. https://doi.org/10.1016/j.devcel.2021.08.015.
20. Hoffmann, P.C., Bharat, T.A.M., Wozny, M.R., Boulanger, J., Miller, E.A., and Kukulski, W. (2019). Tricalbins contribute to cellular lipid flux and form curved ER-PM contacts that are bridged by rod-shaped structures. Dev. Cell *51*, 488–502.e8. https://doi.org/10.1016/j.devcel.2019.09.019.
21. Wong, L.H., Gatta, A.T., and Levine, T.P. (2019). Lipid transfer proteins: the lipid commute via shuttles, bridges and tubes. Nat. Rev. Mol. Cell Biol. *20*, 85–101. https://doi.org/10.1038/s41580-018-0071-5.
22. Jüngst, C., Klein, M., and Zumbusch, A. (2013). Long-term live cell microscopy studies of lipid droplet fusion dynamics in adipocytes. J. Lipid Res. *54*, 3419–3429. https://doi.org/10.1194/jlr.M042515.
23. Shin, K.J., Wall, E.A., Zavzavadjian, J.R., Santat, L.A., Liu, J., Hwang, J.I., Rebres, R., Roach, T., Seaman, W., Simon, M.I., and Fraser, I.D.C. (2006). A single lentiviral vector platform for microRNA-based conditional RNA interference and coordinated transgene expression. Proc. Natl. Acad. Sci. USA *103*, 13759–13764. https://doi.org/10.1073/pnas.0606179103.
24. Dull, T., Zufferey, R., Kelly, M., Mandel, R.J., Nguyen, M., Trono, D., and Naldini, L. (1998). A third-generation lentivirus vector with a conditional packaging system. J. Virol. *72*, 8463–8471. https://doi.org/10.1128/JVI.72.11.8463-8471.1998.
25. Schindelin, J., Arganda-Carreras, I., Frise, E., Kaynig, V., Longair, M., Pietzsch, T., Preibisch, S., Rueden, C., Saalfeld, S., Schmid, B., et al. (2012). Fiji: an open-source platform for biological-image analysis. Nat. Methods *9*, 676–682. https://doi.org/10.1038/nmeth.2019.
26. Kremer, J.R., Mastronarde, D.N., and McIntosh, J.R. (1996). Computer visualization of three-dimensional image data using IMOD. J. Struct. Biol. *116*, 71–76.
27. Mastronarde, D.N., and Held, S.R. (2017). Automated tilt series alignment and tomographic reconstruction in IMOD. J. Struct. Biol. *197*, 102–113. https://doi.org/10.1016/j.jsb.2016.07.011.
28. Mastronarde, D.N. (2005). Automated electron microscope tomography using robust prediction of specimen movements. J. Struct. Biol. *152*, 36–51. https://doi.org/10.1016/j.jsb.2005.07.007.
29. Lawson, C.L., Patwardhan, A., Baker, M.L., Hryc, C., Garcia, E.S., Hudson, B.P., Lagerstedt, I., Ludtke, S.J., Pintilie, G., Sala, R., et al. (2016). EMDataBank unified data resource for 3DEM. Nucleic Acids Res. *44*, D396–D403. https://doi.org/10.1093/nar/gkv1126.
30. Iudin, A., Korir, P.K., Somasundharam, S., Weyand, S., Cattavitello, C., Fonseca, N., Salih, O., Kleywegt, G.J., and Patwardhan, A. (2023). EMPIAR: the electron microscopy public image archive. Nucleic Acids Res. *51*, D1503–D1511. https://doi.org/10.1093/nar/gkac1062.
31. Hoffmann, P.C., Giandomenico, S.L., Ganeva, I., Wozny, M.R., Sutcliffe, M., Lancaster, M.A., and Kukulski, W. (2021). Electron cryo-tomography reveals the subcellular architecture of growing axons in human brain organoids. Elife *10*, e70269. https://doi.org/10.7554/eLife.70269.
32. Russo, C.J., Scotcher, S., and Kyte, M. (2016). A precision cryostat design for manual and semi-automated cryo-plunge instruments. Rev. Sci. Instrum. *87*, 114302. https://doi.org/10.1063/1.4967864.
33. Schaffer, M., Engel, B.D., Laugks, T., Mahamid, J., Plitzko, J.M., and Baumeister, W. (2015). Cryo-focused ion beam sample preparation for imaging vitreous cells by cryo-electron tomography. Bio. Protoc. *5*, e1575. https://doi.org/10.21769/bioprotoc.1575.
34. Hagen, W.J.H., Wan, W., and Briggs, J.A.G. (2017). Implementation of a cryo-electron tomography tilt-scheme optimized for high resolution subtomogram averaging. J. Struct. Biol. *197*, 191–198. https://doi.org/10.1016/j.jsb.2016.06.007.
35. Lord, S.J., Velle, K.B., Mullins, R.D., and Fritz-Laylin, L.K. (2020). SuperPlots: communicating reproducibility and variability in cell biology. J. Cell Biol. *219*, e202001064. https://doi.org/10.1083/jcb.202001064.

CellPress
OPEN ACCESS

STAR★METHODS

KEY RESOURCES TABLE

REAGENT or RESOURCE	SOURCE	IDENTIFIER
Bacterial and virus strains		
One Shot TOP10 Chemically Competent E.coli	Thermo Fisher	Cat#C404003
One Shot Stbl3 Chemically Competent E.coli	Thermo Fisher	Cat#C737303
Chemicals, peptides, and recombinant proteins		
HCS LipidTOX Deep Red Neutral Lipid Stain	Thermo Fisher	Cat#H34477
Oleic acid	Merck	Cat#O3008
Phusion High-Fidelity DNA Polymerase	Thermo Fisher	Cat#F530L
DNA Ligation Kit, Mighty Mix	Takara Bio	Cat#6023
DMEM, high glucose	Thermo Fisher	Cat#11960044
DMEM, high glucose, GlutaMAX	Thermo Fisher	Cat#10569010
Tetracycline-free FBS	Pan Biotech	Cat#P30-3602
Pyruvate	Thermo Fisher	Cat#11360070
L-glutamine	Merck	Cat#59202C
β-mercaptoethanol	Merck	Cat#M3148
Biotin	Merck	Cat#B4639
D-pantothenic acid	Merck	Cat#P5155
Rosiglitazone	Merck	Cat#R2408
3-isobutyl-1-methylxanthine	Merck	Cat#I5879
Dexamethasone	Merck	Cat#D4902
Hygromycin B	Invitrogen	Cat#10687010
MycoAlert mycoplasma detection kit	Lonza	Cat#LT07-418
Gateway LR Clonase II enzyme mix	Thermo Fisher	Cat#11791020
Non-essential amino acids	Merck	Cat#M7145
Lipofectamine LTX with Plus Reagent	Thermo Fisher	Cat#15338100
Fugene 6 transfection reagent	Promega	Cat#E2691
UltraCULTURE serum-free cell culture medium	Lonza	Cat#BE12-725F
RQ1 RNase-Free DNase	Promega	Cat#M610A
LunaScript RT SuperMix Kit	NEB	Cat#E3010L
TaqMan Universal PCR Master Mix	Thermo Fisher	Cat#4304437
Formaldehyde	Merck	Cat#47608
VECTASHIELD Antifade Mounting Medium	Vector Laboratories	Cat#H-1000
Prolong Gold Antifade Mountant	Thermo Fisher	Cat#P36931
Penicillin-Streptomycin	Merck	Cat#P4333
Actrapid human Insulin	Novo Nordisk	N/A
Doxycycline	Clontech	Cat#631311
Puromycin	Cambridge Bioscience	Cat#P025-P026
Polybrene	Merck Millipore	Cat#TR-1003-G
Sodium pyruvate	Merck	Cat#S8636
Critical commercial assays		
RNeasy Mini Kit	QIAgen	Cat#74106
Deposited data		
Representative tomogram of Cidec-EGFP expressing HeLa cells	This paper; deposited at EMDB	EMD-16455
Representative tomogram of Cidec expressing HeLa cells	This paper; deposited at EMDB	EMD-16454

(Continued on next page)

Continued

REAGENT or RESOURCE	SOURCE	IDENTIFIER
Raw tilt images and tomograms of Cidec-EGFP expressing HeLa cells	This paper; deposited at EMPIAR	EMPIAR-11394
Raw tilt images and tomograms of Cidec expressing HeLa cells	This paper; deposited at EMPIAR	EMPIAR-11393
Live cell fluorescence images of HeLa cells expressing Cidec constructs	This paper; deposited at Zenodo	https://doi.org/10.5281/zenodo.7534719
Experimental models: Cell lines		
HeLa transduced with EGFP	This paper	N/A
HeLa transduced with Cidec untagged	This paper	N/A
HeLa transduced with Cidec-EGFP	Ader et al.[16]	N/A
HeLa transduced with Cidec-SUMOstar	This paper	N/A
Cidec null mouse embryonic fibroblasts (MEFs)	Gift from Masato Kasuga Nishino et al.[2]	N/A
Cidec null MEF transduced with Cidec-EGFP	This paper	N/A
Cidec null MEF transduced with EGFP	This paper	N/A
BOSC 23 retroviral packaging cells	ATCC	Cat#CRL-11270
HEK293T cells	ECACC	Cat#12022001
Oligonucleotides		
Cidec	Thermo Fisher	Mm01184685_g1
GAPDH	Thermo Fisher	Hs02758991_g1
Recombinant DNA		
Gateway Entry vector, pEN-Tmcs	Gift from Iain D. C. Fraser Shin et al.[23]	Addgene no. 25751
pEGFPN3 vector	Clontech	Cat#6080-1
Gateway Destination vector, pSLIK-hygromycin	Gift from Iain D. C. Fraser Shin et al.[23]	Addgene no. 25737
pMDLg/pRRE	Gift from Didier Trono Dull et al.[24]	Addgene no.12251
pRSV-Rev	Gift from Didier Trono Dull et al.[24]	Addgene no.12253
pVSV-G (also known as pMD2.G)	Gift from Didier Trono	Addgene no.12259
pBABE-mPPARγ2	This paper	N/A
pBABE-EGFP	This paper	N/A
Software and algorithms		
Prism	GraphPad	N/A
Fiji	Schindelin et al.[25]	https://imagej.net/software/fiji/
Bitplane Imaris software version 9.6.0	Oxford Instruments	N/A
MATLAB	MathWorks	N/A
IMOD	Kremer et al., Mastronarde et al.[26,27]	https://bio3d.colorado.edu/imod/
SerialEM	Mastronarde et al.[28]	https://bio3d.colorado.edu/SerialEM/
Custom MATLAB scripts for analysis of distances between LDs	This paper	https://gitlab.com/jboulanger/bioimglab
Custom MATLAB scripts for analysis of transfer kinetics in live FM	This paper	https://gitlab.com/jboulanger/ldtrack
Other		
Quantifoil holey carbon EM grids (gold, 200 mesh, R2/2)	www.quantifoil.com	N/A

RESOURCE AVAILABILITY

Lead contact

Request for resources and further information should be directed to and will be fulfilled by the lead contact, Wanda Kukulski (wanda.kukulski@unibe.ch).

Materials availability

Cell lines generated in this study are available from the lead contact upon request with a completed Materials Transfer Agreement.

Data and code availability

Data and code are publicly available as follows: Representative electron cryo-tomograms are deposited at the Electron Microscopy Data Bank (EMDB).[29] Raw tilt images and tomographic reconstructions of analyzed electron cryo-tomograms are deposited at the Electron Microscopy Public Image Archive (EMPIAR).[30] Live fluorescence microscopy data is deposited at Zenodo. All original code has been deposited on gitlab. All accession numbers, DOIs and URLs are listed in the key resources table. Any additional information required to reanalyze the data reported in this paper is available from the lead contact upon request.

EXPERIMENTAL MODEL AND SUBJECT DETAILS

Cell lines

Primary *Cidec* null MEFs were cultured at 37°C, 5% CO_2 in high glucose DMEM (Thermo Fisher, 11960044) supplemented with 10% Tetracycline-free heat-inactivated FBS (Pan Biotech, P30-3602), 100 U/mL penicillin, 100 μg/mL streptomycin (Merch, P4333), 2 mM L-glutamine (Merck, 59202C), 1x non-essential amino acids (Merck, M7145), 1 mM sodium pyruvate (Merck, S8636), and 50 μM β-mercaptoethanol (Merck, M3148). The MEFs were immortalised by transfection of 2 μg of simian virus 40 large T-antigen-expressing vector employing Fugene 6 transfection reagent (Promega, E2691) followed by five rounds of a 1 in 10 split to achieve 1/100,000-fold splitting. Untransfected primary MEFs that underwent the 1/100,000-fold splitting were used as a control to ensure that all surviving cells were immortalised. Immortalised *Cidec* null MEFs were then transduced with pBABE-mPPARγ2 to ensure they had the potency to differentiate into adipocytes. BOSC 23 retroviral packaging cells were ~70% confluent when transfected with 12 μg of pBABE-mPPARγ2 or pBABE-EGFP plasmid DNA using Fugene 6 transfection reagent (Promega, E2691). Media containing secreted retrovirus was collected 72 h post-transfection and filtered through 0.45 μm syringe filters. The filtered retroviral stocks were used to transduce ~50–60% confluent immortalised *Cidec* null MEFs with the addition of 12 μg/mL polybrene (Merck Millipore, TR-1003-G). *Cidec* null MEFs transduced with pBABE-EGFP acted as an indicator of transduction efficiency. Puromycin (Cambridge Bioscience, P025-P026) selection was initiated 24 h post-transduction at a concentration of 4 μg/mL.

To induce differentiation of *Cidec* null MEFs into adipocytes, cells were grown to 2-days post-confluency, then induced to differentiate in culture medium supplemented with 8 μg/mL biotin (Merck, B4639), 8 μg/mL D-pantothenic acid (Merck, P5155), 1 μM rosiglitazone (Merck, R2408), 0.5 mM 3-isobutyl-1-methylxanthine (Merck, I5879), 1 μM dexamethasone (Merck, D4902) and 1 μM insulin (Novo Nordisk). Two days thereafter, culture medium was changed to contain 8 μg/mL biotin, 8 μg/mL D-pantothenic acid, 1 μM rosiglitazone and 1 μM insulin. From day 4 of differentiation onwards, culture medium was changed every 2 days until the cells were used for experiments.

HeLa Doxycycline-inducible stable cell lines were grown as an adherent culture at 37°C, 5% CO_2 in a high glucose DMEM media containing pyruvate (Thermo Fisher, 11360070), GlutaMAX (Thermo Fisher, 10569010). The media was additionally supplemented with 10% Tet-approved heat-inactivated FBS, 10 mM HEPES pH 7.2 and 0.2 mg/mL hygromycin B (Invitrogen, 10687010). Cell lines were regularly tested for mycoplasma infection using the MycoAlert mycoplasma detection kit (Lonza, LT07-418). The HeLa cell line expressing Cidec-EGFP was authenticated by Eurofins using PCR-single locus technology.

METHOD DETAILS

Cloning

EGFP and mouse *Pparγ2* cDNA were amplified by PCR using Phusion High-Fidelity DNA Polymerase (Thermo Fisher, F530L) and each cloned into pBABE-puro retroviral expression vector using SnaBI and SalI restriction sites. All Cidec constructs used in this study contain the *Mus musculus* Cidec sequence. Mouse Cidec cDNA was amplified by PCR using Phusion High-Fidelity DNA Polymerase (Thermo Fisher, F530L). The untagged Cidec transcript flanked by SacII and NotI restriction sites at the amino- and carboxyl-terminus, respectively, was cloned into Gateway Entry vector, pEN-Tmcs, using a DNA Ligation Kit, Mighty Mix (Takara Bio, 6023) and transformed into One Shot TOP10 Chemically Competent *E.coli* (Thermo Fisher, C404003). For EGFP tagging at the carboxyl-terminus, a SacII and BamHI restriction site flanked Cidec sequence was inserted into a pEGFPN3 vector (Clontech, 6080-1). For SUMOstar tagging at the carboxyl-terminus, a BamHI and SalI restriction site flanked Cidec sequence, a SalI and NotI flanked SUMOstar sequence, and a NotI and XbaI flanked Twin-strep-tag sequence were inserted into a pACEMam1 vector by enzyme restriction cloning. The sequence-verified insert was subcloned into a pEN-Tmcs (Addgene, 25751)[23] vector using SacII and NotI restriction sites. In order to generate expression clones, sequence-verified inserts along with Tet-responsive elements flanked by *att*L sites in the Gateway Entry vector were recombined into a Gateway Destination vector,[23] pSLIK-hygromycin containing *att*R sites using Gateway LR Clonase II enzyme mix (Thermo Fisher, 11791020). Recombination reactions were transformed into One Shot Stbl3 Chemically Competent *E.coli* (Thermo Fisher, C737303).

Generation of *Cidec* null MEF and HeLa cell lines with Doxycycline-inducible expression of Cidec constructs

Cidec null MEFs and HeLa cell lines with Doxycycline-inducible expression of Cidec (untagged, SUMOstar- and EGFP-tagged Cidec) were generated by lentiviral transduction. To generate lentiviruses, HEK293T cells at approximately 70% confluency were transfected using Lipofectamine LTX with Plus Reagent (Thermo Fisher, 15338100) according to the manufacturer's protocol. A typical transfection reaction included 7.5 μg pMDLg/pRRE (Addgene, 12251), 7.5 μg pRSV-Rev (Addgene 12253),[24] 5 μg pVSV-G (Addgene, 12259), 1 μg pEGFP and 10 μg pSLIK-hygromycin plasmid DNA (with integrated untagged, SUMOstar- and EGFP-tagged Cidec

cDNA sequences). 24 h post-transfection, the culture medium was removed and the cells were replenished with UltraCULTURE serum-free cell culture medium (Lonza, BE12-725F). Medium containing secreted lentivirus was collected every 24 h for a total of 72 h and stored at 4°C. Lentivirus-containing media was centrifuged at 2000 g at 4°C for 20 min and the supernatant was filtered through 0.45 μm syringe filters.

To concentrate the lentiviral supernatant, Centricon Plus-70 Centrifugal Filters (Merck Millipore, UFC710008) were used according to the manufacturer's protocol. Sample filter cups were pre-rinsed with PBS and lentiviral supernatant was loaded onto the sample filter cups, sealed and centrifuged at 3500 g for 30 min at 15°C. To recover the concentrated lentivirus, the concentrate collection cups were inverted and placed on top of the sample filter cups, and then centrifuged at 3500 g for 10 min at 15°C. The concentrated lentivirus was either used to transduce *Cidec* null MEFs and HeLa cells at 2 to 3 different viral titers, or aliquoted into cryovials for long term storage at −80°C. Cells were selected 24 h post-lentiviral transduction using 200 μg/mL of hygromycin B (Invitrogen, 10687010). Non-lentivirus transduced cells were used as a control for antibiotic selection. The HeLa cell line expressing Cidec-EGFP has been reported by us before.[16,31]

Determination of mRNA transcript levels of *Cidec* constructs in HeLa cells

In order to determine and achieve comparable mRNA expression levels of *Cidec* in untagged, SUMOstar- and EGFP-tagged HeLa cell lines, Doxycycline (Clontech, 631311) of 0.5, 1.0 or 2.0 μg/mL was added to seeded cells in the presence of 200 μM of oleic acid (Merck, O3008). 24 h post-induction, RNA was harvested using an RNeasy Mini Kit (QIAgen, 74,106) by following the manufacturer's protocol. 400 ng of RNA was treated with 1 unit of RQ1 RNase-Free DNase (Promega, M610A) at 37°C for 30 min and was inactivated by RQ1 DNase Stop Solution at 65°C for 10 min. To generate cDNA standards and a negative control for reverse transcription, 1 μg of pooled RNA was prepared. RNA was reverse transcribed into cDNA using a LunaScript RT SuperMix Kit (NEB, E3010L) by following the manufacturer's protocol, using thermocycling conditions as follows: 25°C for 2 min, 55°C for 10 min and 95°C for 1 min. cDNA was diluted 10x and qPCR was performed on an Applied Biosystem QuantStudio 7 Flex Real-Time PCR System. A typical TaqMan qPCR reaction included 1x TaqMan Universal PCR Master Mix (Thermo Fisher, 4304437) and 1x TaqMan Gene Expression Assay, in which *GAPDH* was used as a housekeeping gene (Cidec: Mm01184685_g1; GAPDH: Hs02758991_g1). mRNA levels were normalized to the levels in untagged *Cidec* line untreated with Doxycycline. From this, it was determined that 0.5 μg/mL of Doxycycline in untagged Cidec and Cidec-EGFP lines induced comparable mRNA expression levels of *Cidec* as 2.0 μg/mL of Doxycycline in the Cidec-SUMOstar line. Thus, these Doxycycline concentrations were used for the described fixed and live cell imaging experiments.

Fixed cell imaging

HeLa cells and MEF cells were cultured in the corresponding media mentioned in the section "cell lines". Cells were seeded onto ethanol-treated glass coverslips in 12-well plates. *Cidec* null MEFs were differentiated into mature adipocytes according to the protocol above. 1 μg/mL of Doxycycline was either added or omitted throughout the course of differentiation. An hour before fixation, LDs were stained with 1x HCS LipidTOX Deep Red Neutral Lipid Stain (Thermo Fisher, H34477) at 37°C. To study the effects of Cidec on LD enlargement at a fixed time point in HeLa cells expressing Doxycycline-inducible Cidec constructs, the day after seeding cells were washed twice with PBS and loaded with 200 μM oleic acid and various Doxycycline concentrations (0.5–2 μg/mL) in order to achieve comparable Cidec transcript levels (see above). 23 h post-Doxycycline induction, LDs were stained with 1x HCS LipidTOX Deep Red Neutral Lipid Stain for an hour at 37°C.

Cells were fixed with 4% (v/v) formaldehyde (Merck, 47608) diluted in PBS for 15 min at room temperature. Cells were then washed 3 times for 5 min with PBS and mounted using VECTASHIELD Antifade Mounting Medium (Vector Laboratories, H-1000) or Prolong Gold Antifade Mountant with DAPI (Thermo Fisher, P36931). For *Cidec* null MEF-derived adipocytes, 2-dimensional images were acquired using a Leica SP8 confocal microscope. EGFP and LipidTOX Deep Red Neutral Lipid Stain were excited at 488 and 637 nm, and emission signals were collected at 495–535 and 645–700 nm, respectively. For HeLa cell lines, 3-dimensional images were acquired using a Leica SP8 confocal microscope at 0.3–0.4 μm z sections. EGFP and LipidTOX Deep Red Neutral Lipid Stain were excited at 488 nm and 637 nm, and emission signals were collected at 495–550 nm and 645–690 nm, respectively. Experiments were repeated three times for *Cidec* null MEFs-derived adipocytes with 25 cells being analyzed for LD volume in each experiment and three times for HeLa cells with at least 11 cells being analyzed in each experiment.

Analysis of LD sizes and number in fixed cell fluorescence images

In 2-dimensional images of fixed *Cidec* null MEFs-derived adipocytes, LD sizes were derived by measuring LD diameters using Fiji software[25] based on LipidTOX Deep Red dye staining. A line was drawn across the LDs on focal planes and these measurements were used to calculate LD volumes by assuming that LDs are spherical in shape. In images of fixed HeLa cells, LD sizes and numbers were determined by using Bitplane Imaris software version 9.6.0 (Oxford Instruments), based on LipidTOX Deep Red staining. The 3-dimensional images were segmented by 'Spots' creation wizard with 'Different Spot Sizes (Region Growing)' algorithm setting enabled. 'Estimated XY Diameter' was set between 0.6 and 2.0 μm with 'Background Subtraction' enabled. Images were further filtered using 'Quality' filter type of at least 2.7 and 'Spot Region Type' of 'Absolute Intensity'. Diameters of the 'Spot Regions' were determined from 'Region Border' at thresholds of 16–35. LD numbers per cell were obtained based on the number of spots detected.

Live cell imaging

HeLa cells were seeded onto 35 mm imaging dishes with a polymer coverslip bottom (Ibidi, 81156). 24 h before live cell imaging was performed, 200 μM oleic acid was added to cells in culture media supplemented with 10% Tetracycline-free FBS (Pan Biotech, P30-3602), 2 mM L-glutamine (Merck, 59202C), 100 units/mL penicillin and 0.1 mg/mL streptomycin (Merck, P4333) and 200 μg/mL Hygromycin B (Merck Millipore, 400052). Two hours before imaging of HeLa cells with Doxycycline-inducible expression of untagged Cidec or Cidec-SUMOstar, 0.5 or 2 μg/mL of Doxycycline, respectively, was added to the cells in the presence of 1x HCS LipidTOX Deep Red Neutral Lipid Stain. In the case of Cidec-EGFP, 0.5 μg/mL of Doxycycline was added 24 h before imaging. Live cell imaging was performed on a Leica SP8 confocal microscope in a chamber maintained at 37°C with 5% CO_2, using a 63x objective with NA = 1.4, and using an additional two-fold or three-fold zoom on the microscope level. LipidTOX Deep Red Neutral Lipid Stain was excited at 633 nm, and the emission signal was collected 640–700 nm. For HeLa cells expressing untagged Cidec or Cidec-SUMOstar, multiple positions of 3-dimensional images were acquired at 20 s intervals at 0.4 μm z sections for at least 2 h, while for Cidec-EGFP, 3-dimensional images were acquired at 2.5-min intervals for at least 20 h. During all acquisitions of untagged Cidec and Cidec-SUMOstar data, a three-fold zoom was used, resulting in a pixel size of 120 nm, while in 3 out of 4 data acquisition sessions for Cidec-EGFP, a two-fold zoom on the microscope was used, resulting in a pixel size of 180 nm, as compared to 120 nm for all other data. The experiments were performed on different days with cells cultured individually.

Quantification of frequency of active lipid transfer events

To determine the frequency of two juxtaposed LDs being engaged in active lipid transfer, 100 pairs of contacting LDs in oleic acid fed HeLa cells expressing Cidec-EGFP or untagged Cidec were analyzed from live cell fluorescence images. Maximum projection images generated by Fiji from 3D stacks were used. For each LD analyzed, the average of two or more measured diameters was used to derive the volume of the lipid donors, assuming that LDs are spherical in shape. A change in LD volume over the imaging period was considered as a lipid transfer event.

Manual analysis of lipid transfer rate

To determine the rate of neutral lipid transfer, maximum projection images were generated from the 3D stacks using Fiji[25] and the diameters of donor LDs were measured at least at two different positions using Fiji. The average of the two or more measured diameters was used to derive the volume of the lipid donors. The change of the lipid donor volume over time (Δvolume/Δtime) was calculated by assuming that LDs are spherical in shape. In all cases, the rate of neutral lipid transfer was determined for donors that underwent active lipid transfer and were tethered to a single acceptor. In the case of Cidec-EGFP, only LD pairs with enriched Cidec-EGFP at the LD interfaces were analyzed.

Semi-automated analysis of lipid transfer rate

Kinetics of neutral lipid transfer were analyzed with a custom-made MATLAB (MathWorks) script. Movies were acquired as described above and individual LDs were further segmented out and tracked over the course of the 3D image sequence. In the script, an event at time t_{max} is defined as a merging of two tracks into a single one, and the donor LD is identified as the LD whose volume will vanish at t_{max}. The jump in the volume of the donor at the end of the event corresponds to the volume of the donor being equal to the volume of the acceptor once transfer is completed. The evolution of the volume of the donor droplet over time is modeled as $V_d(t) = V_{d0}(1 - \exp(R_d\ (t - t_{max}))$ where V_{d0} is the initial volume of the donor and R_d is the transfer rate (R_{Donor}). Similarly, the volume of the acceptor droplet is modeled as $V_a(t) = V_{a0} + (V_{amax} - V_{a0}) \times \exp(R_a\ (t - t_{max}))$ where V_{a0} and V_{amax} are respectively the initial and final volume of the acceptor and R_a is the transfer rate ($R_{Acceptor}$). In both cases, a least-square procedure allows to retrieve the 5 parameters for each event. Each LD pair is considered as an individual event. To exclude outliers, corresponding to LDs in contact with more than one other LD, the rate R_d for the donor was plotted against the rate R_a for the acceptor for each event. We considered only those transfer events for which $|\log_{10}R_a / \log_{10}R_d - 1| < 0.4$, thereby excluding events likely to be involved in multiple transfers.

Sample preparation for cryo-FM and cryo-FIB milling

HeLa Doxycycline-inducible stable cell lines were grown for approximately 48 h on holey carbon gold EM grids (200 mesh, R2/2, Quantifoil) prior to plunge freezing. 24 h after seeding, HeLa cells expressing Cidec-EGFP were fed with 0.2 mM oleic acid (Sigma, OA O3008) and induced with 1 μg/mL Doxycycline for Cidec-EGFP expression or grown further in the absence of Doxycycline. 16 h later these cells were stained for 1 h with HCS LipidTOX Deep Red Neutral Lipid Stain (Thermo Fisher, H34477) and plunge frozen. HeLa cells expressing untagged Cidec were grown for 24 h and then fed with 0.2 mM oleic acid. 24 h later, these cells were induced with 1 μg/mL Doxycycline for Cidec expression. 30 min post-induction, cells were stained for 1 h with LipidTOX Deep Red dye and 1 h 30 min post-induction, cells were plunge frozen. Plunge freezing was performed with a home-built manual plunger and cryostat.[32] To this end, grids were manually back side blotted with Whatman filter paper No 1 and immediately vitrified in liquid ethane. Grids were screened at −195°C for ice quality and for regions featuring cells of interest by cryo-FM in a Leica EM cryo-CLEM system (Leica Microsystems) in a humidity-controlled room. The system used to image Cidec-EGFP and untagged Cidec expressing cells was equipped with an HCX PL APO 50x cryo objective with 0.9 NA (Leica Microsystems), an Orca Flash 4.0 V2 sCMOS camera (Hamamatsu Photonics), a Sola Light Engine (Lumencor), an L5 filter set (Leica Microsystems) for the detection of EGFP and a Y5 filter set (Leica

Microsystems) for LipidTOX Deep Red detection. The system used to image cells not expressing Cidec-EGFP was equipped with an HCX PL APO 50x cryo objective with 0.9 NA (Leica Microsystems), a DFC9000 GT sCMOS camera (Leica Microsystems), an EL 6000 light source (Leica Microsystems), and a Y5 filter set (Leica Microsystems) for LipidTOX Deep Red detection. 1.5 × 1.5 mm montages of the central part of the grids were taken. These montages were later manually correlated with scanning electron beam micrographs and served for identifying the regions of interest for lamella preparation by cryo-FIB milling. Z-stacks in 1 μm steps were acquired of regions of interest corresponding to cells with enlarged LDs and Cidec-EGFP accumulation at LD-LD interfaces.

Cryo-FIB milling

Thin lamellae of HeLa cells expressing either Cidec-EGFP or untagged Cidec or of cells not induced for expression of Cidec-EGFP were generated by cryo-FIB milling performed with a Scios DualBeam FIB/SEM (FEI) equipped with a Quorum stage (PP3010T) or an Aquilos 2 Cryo FIB (Thermo Scientific) using a similar procedure as published before.[33] In the Scios, grids were coated with organometallic platinum using a gas injection system for 30 s at 13 mm working distance and 25° stage tilt. In the Aquilos, used only for lamella preparation of non-induced cells, grids were first splutter coated at 30 mA, 0.1 kV for 15 s. Subsequently the grids were subjected to GIS coating for 1 min at a working distance of 10 mm, which corresponds to a platinum layer of approximately 1 μm. In both systems, the electron beam was used for locating the cells of interest at 5 kV voltage and 13 pA current and for imaging to check progression of milling at 2 kV voltage and 13 pA current. Milling with the ion beam was performed stepwise. For rough milling the voltage was kept at 30 kV throughout all steps and milling was performed simultaneously from the top and the bottom of the lamella. The current and stage position were adjusted as follows: 1) 1 nA, 35° stage tilt until a lamella thickness of 20 μm; 2) 0.5 nA, 25° tilt until 12 μm; 3) 0.3 nA, 17° tilt until 3 μm; and 4) 0.1 nA, 17° tilt until 1 μm. For the fine milling steps, the voltage was lowered to 16 kV and the current to 23 pA. The stage was first tilted to 18° and the lamella was milled only from the top, then the stage was tilted to 16° and the lamella was milled only from the bottom. These two steps resulted in a lamella thickness of approximately 0.5 μm. Finally, the stage was tilted back to 17° and the lamella was milled simultaneously from the top and the bottom to a target thickness below 0.3 μm.

Electron cryo-tomography (cryo-ET)

Cryo-ET data of FIB-milled cells was acquired on three different Titan Krios microscopes (Thermo Scientific), all equipped with a K2 direct electron detector and a BioQuantum energy filter (Gatan). SerialEM was used for acquisition.[28] To identify the positions of the lamellae, low-magnification montages of the central part of the grid were acquired , with the detector operated in linear mode. Then2.3, 5.1 or 5.5 nm pixel size montages of individual lamellae were taken and used for finding interfaces between LDs. Tilt series were acquired from 0° to ±60° (maximum) using a dose-symmetric acquisition scheme,[34] 1° increment and a pixel size of 3.7, 3.5 or 3.4 Å. The detector was operated in counting mode. Images were acquired in a tilt group size of 4. The target defocus was set to −5 μm. The dose per tilt series image was adjusted to 1–1.2 e^-/A^2 and the target dose rate at the detector was kept around 4 e^-/px/s. For a subset of the tilt series, we used exposure fractionationation into 3 frames per tilt image. Alignment of exposure fractionated frames was performed in IMOD using alignframes. Tilt series were aligned in IMOD using patch tracking.[26,27] Final tomograms were reconstructed at 7.5, 7.1 or 6.7 Å pixel size using SIRT reconstruction with 10 iterations. For presentation in figure panels and movies, a median filter was applied to virtual slices. The cryo-EM data presented in the manuscript for Cidec-EGFP was obtained from 30 cells expressing Cidec-EGFP, plunge-frozen on at least 8 different days, and 15 tomograms containing LD interfaces were acquired on 10 of these cells. The cryo-EM data presented for Cidec was obtained from 15 cells expressing Cidec, frozen on 3 days, and 7 tomograms containing LD interfaces were acquired on 7 of these cells. The cryo-EM data presented of cells not expressing Cidec-EGFP was obtained from 5 cells frozen on 2 different days, and one representative tomogram, showing an LD but not containing LD interfaces, is included in the manuscript. Three of the tomograms of cells expressing Cidec-EGFP were previously used in unrelated projects published before.[16,31]

Analysis of distances between lipid droplets

Distance measurements were performed in electron cryo-tomograms using a custom-made MATLAB (MathWorks) script. The mrc volume was converted to 8-bit tif images, wich were subsequently subjected to a median filter with radius 2 px in Fiji to enhance visibility of the monolayers. Control points along each monolayer were manually defined at positions where the monolayers were best visible. The monolayers were traced every 7.5 or 7.1 Å in z direction through the tomographic volume (see Figure S4B). Based on these points, the surface of each monolayer was interpolated on a 2 nm regular grid. The distance between points of the interpolated surfaces were computed, and various descriptive statistics were obtained. In particular, heatmaps of the distances (Figures S4C and S4D), minimum and maximum distance as well as the mean, median and the standard deviation of the distances were calculated. Each interface between an LD pair is considered as an individual event. Distances measured between the regularly spaced points of the interpolated surfaces were plotted as scatterplots for each individual interface (Figures S4E and S4F). Calculated medians of the distance at these interfaces were plotted as a scatterplot. For the analysis, we chose to use medians, because the distance measurements are not Gaussian distributions.

Determination of lipid droplet diameters

LD diameters used to calculate the ratios shown in Figure 3D were estimated in IMOD applying imodcurvature to a model consisting of points picked along the LD monolayer in a single virtual tomographic slice. We assumed that LDs are spherical in shape as in the

analysis of FM images (see above). If the equatorial plane of the LD appeared to be included in the tomogram, a single imodcurvature readout from the corresponding virtual slice was used to estimate the LD diameter. If the LD segment contained in the tomographic volume did not include the equatorial plane, two imodcurvature radii a and b were determined on two different virtual slices spaced in z-direction by z nm. The LD radius r was then calculated using the formula: $r = \sqrt{\left(a^2 + \left(\frac{a^2 - b^2 - z^2}{2 * z}\right)^2\right)}$. In one case of a large LD forming two interfaces with smaller LDs, the radius of the large LD could not be estimated because the fraction of the LD contained in the tomogram was too small. The two corresponding interfaces were therefore excluded from the analysis in Figure 3D.

Determination of degree of monolayer deformation

The extent of the disturbance of the monolayers analyzed in Figure 3E was assessed only in tomograms of optimal quality and cellular preservation. Due to insufficient vitrification of the areas of interest, a total of 5 tomograms were excluded. The exclusion concerns 4 interfaces from 2 Cidec-EGFP tomograms and 5 interfaces from 3 untagged Cidec tomograms. The waviness of the monolayers at the LD interface was assessed qualitatively and attributed to one of three arbitrary classes: minimal, intermediate, and maximal deformations. Intermediate and maximal deformations were attributed if the extend of monolayer deformation appeared higher in the interface than in the monolayer outside of the interface.

QUANTIFICATION AND STATISTICAL ANALYSIS

All p values reported in the text are based on statistical tests performed on the data presented in the corresponding Figure panels. In all applicable instances, we plotted data from independent experiments in different colors, similarly to ‘superplots’ (Figures 1A, 1B, 4B–4D).[35] All statistical tests and plots were done in Graphpad Prism. Statistical tests have been applied on the data shown as follows. The datasets in Figures 1A and 1B did not pass normality tests. We therefore used a non-parametric two-tailed Mann-Whitney test to compare -Dox versus + Dox. In Figure 3D the dataset with high enough n passed a normality test hence we used an ordinary one-way ANOVA test to compare all other morphologies against ‘indentation’ (comparison against a control column). In Figure 3F, we used an unpaired parametric two-tailed t test to compare two independent groups, which are sampled from Gaussian distributions. The datasets in Figures 4B and 4C did not pass normality tests and we therefore used a Mann-Whitney test to compare two groups: -Dox versus + Dox for each individual construct. As we assessed the effect only in one direction (increase in mean LD volume in 4B and decrease in mean LD number in 4C), we chose one-tailed Mann-Whitney tests for the two-group-comparisons. We used a non-parametric Kruskal-Wallis test to perform multiple comparisons between all constructs in both 4B and 4C (+Dox). The datasets shown in Figure 4D did not pass normality tests and consequently we used a non-parametric Kruskal-Wallis test to perform multiple comparisons of three independent groups. In Figure 5F, two independent groups with non-gaussian distributions were compared using a non-parametric two-tailed Mann-Whitney test. In Figure S3A, a Chi square contingency test was used to compare two groups of categorical variables.

Cell Reports, Volume *42*

Supplemental information

The architecture of Cidec-mediated interfaces between lipid droplets

Iva Ganeva, Koini Lim, Jerome Boulanger, Patrick C. Hoffmann, Olivia Muriel, Alicia C. Borgeaud, Wim J.H. Hagen, David B. Savage, and Wanda Kukulski

Supplemental Figures and Legends

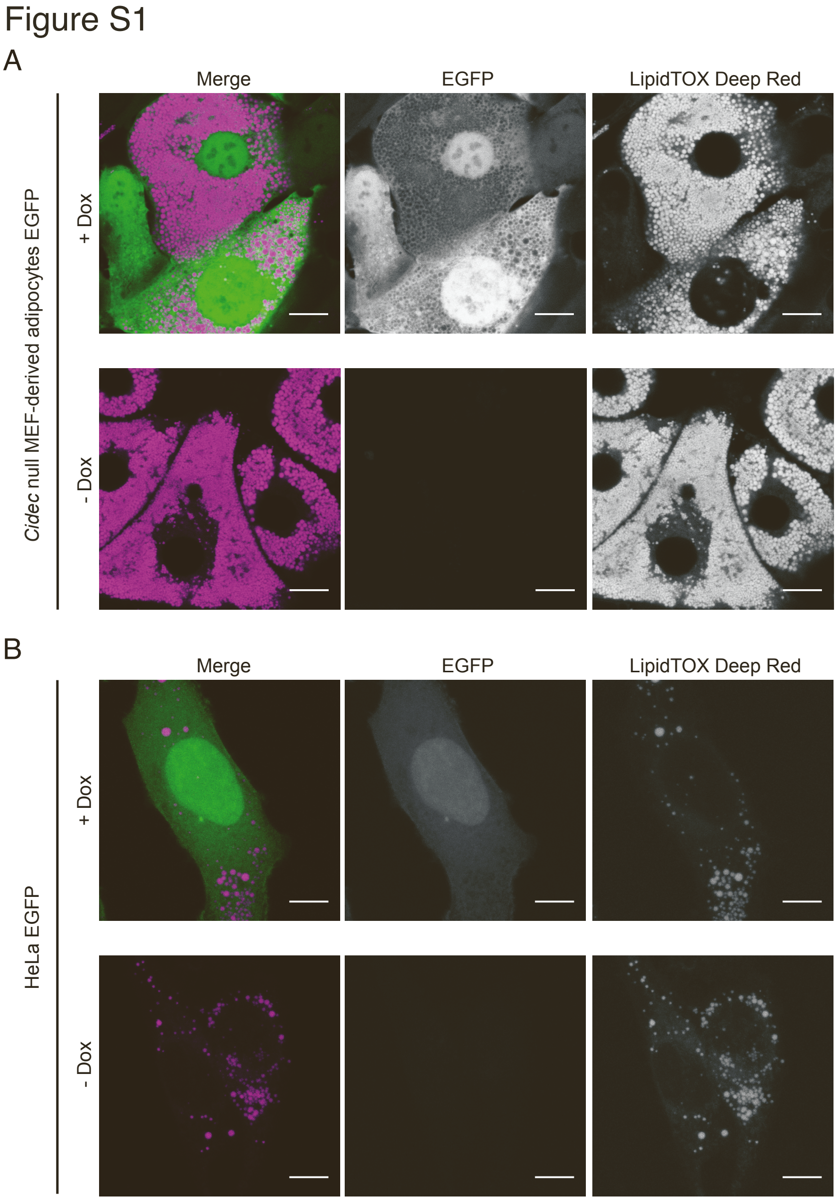

<u>Supplemental Figure S1: Expression of cytosolic EGFP.</u> Related to Figure 1. **A)** Fluorescence microscopy (FM) of fixed *Cidec* null MEF-derived adipocytes inducibly expressing cytosolic EGFP (green). Lipid droplets (LDs) were stained with LipidTOX Deep Red dye (magenta). *Cidec* null MEF-derived adipocytes were induced with Doxycycline (upper panels, + Dox) or not induced (lower panels, -Dox) for expression of cytosolic EGFP throughout the course of differentiation. **B)** FM of fixed HeLa cells inducibly expressing cytosolic EGFP. LDs were stained with LipidTOX Deep Red. HeLa cells were induced with Doxycycline (upper panels, +Dox) or not induced (lower panels, -Dox) for expression of cytosolic EGFP. Images in B were acquired on fixed HeLa cells 24 hours after the corresponding treatment (+Dox/ -Dox). Scale bars: 10 μm.

Figure S2

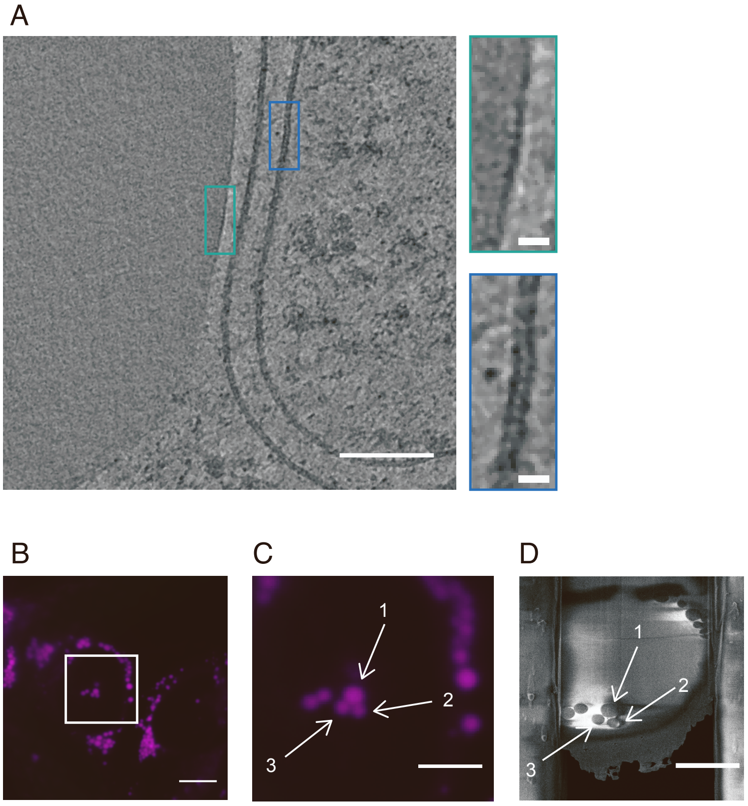

<u>Supplemental Figure S2: Cryo-correlative light and electron microscopy (cryo-CLEM) of HeLa cells expressing Cidec constructs.</u> Related to Figures 2 and 3. **A)** Comparison of the appearance of a monolayer with the appearance of a bilayer. Virtual slice through an electron cryo-tomogram of a HeLa cell inducibly expressing Cidec-EGFP. Green box shows a magnified view of a monolayer, blue box shows a magnified view of a bilayer. **B and C)** Cryo-FM of plunge-frozen HeLa cells induced for expression of untagged Cidec, grown on a cryo-EM grid. Region for cryo-focused ion beam (cryo-FIB) milling was chosen based on LD size, indicating enlargement. **C)** Magnified view of the region shown in the white box in B. White arrows indicate LDs. **D)** Cryo-scanning electron microscopy (cryo-SEM) overview image of a lamella generated from the HeLa cell shown in B and C, thinned by cryo-FIB milling. Interacting LDs identified by cryo-FM are visible in the resulting lamella (white arrows). Scale bars: 100 nm in A (large image), 10 nm in A (magnified views), 10 μm in B, 5 μm in C and D.

Figure S3

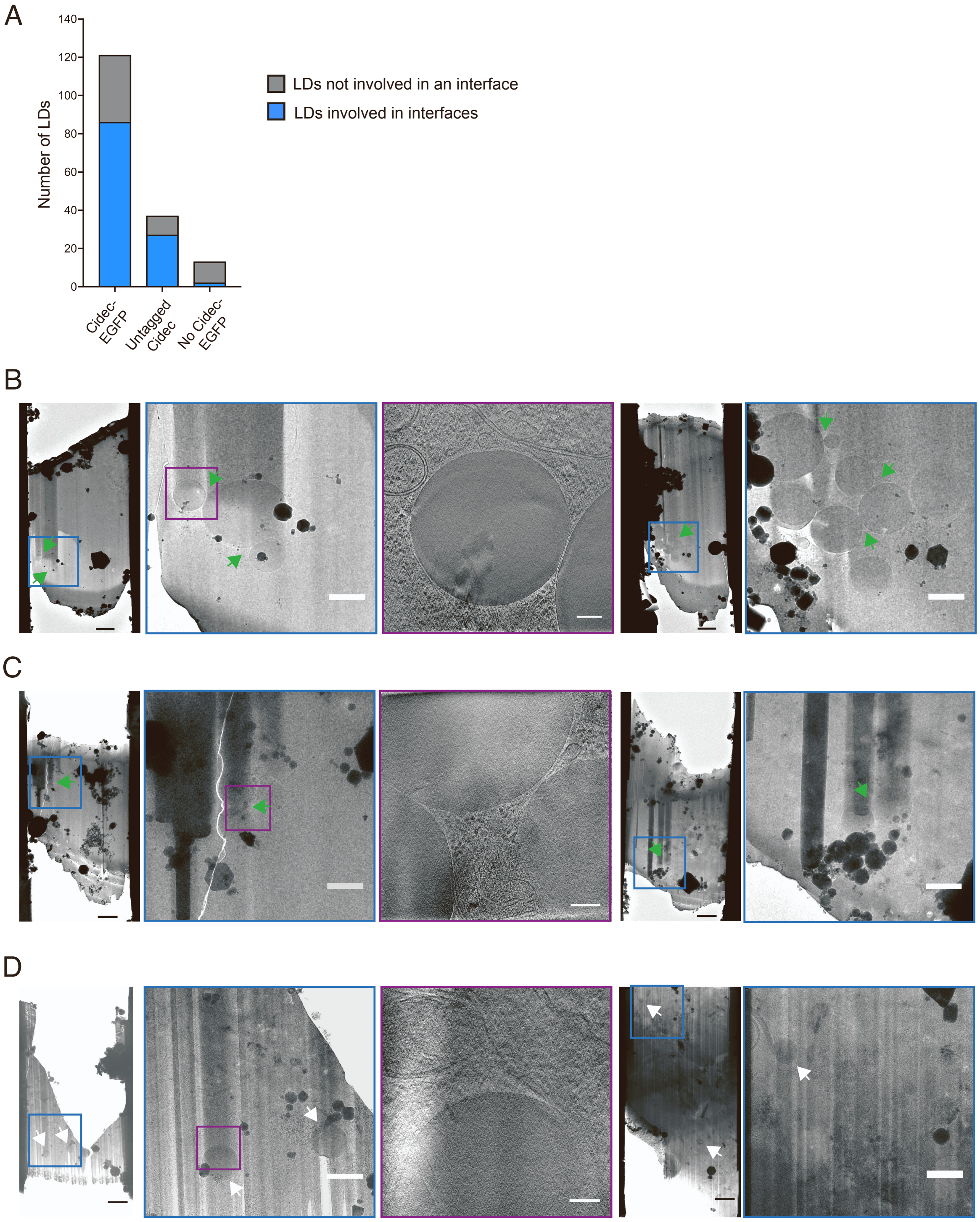

<u>Supplemental Figure S3: Proximity of LDs to each other in cells expressing Cidec-EGFP, untagged Cidec, and non-induced cells.</u> Related to Figures 2 and 3. HeLa cells were fed with oleic acid and either induced with Doxycycline for Cidec-EGFP or Cidec expression, or not induced for Cidec-EGFP expression by not adding Doxycycline. Plunge frozen cells were subjected to cryo-FIB milling and imaged by cryo-EM. **A)** Number of LDs visualised by 2D cryo-EM imaging of lamellae of cells expressing

Cidec-EGFP, untagged Cidec, or not expressing Cidec-EGFP. The LDs were classified into two groups, based on whether they were in contact with at least one other LD or not. 71% of the LDs (n=121) in lamellae of Cidec-EGFP expressing cells and 73% of LDs (n=37) in lamellae of Cidec expressing cells were forming interfaces in contrast to 15% of LDs (n=13) in lamellae of cells not expressing Cidec-EGFP (P=0.0002 for Chi-square contingency test). Note that all lamellae containing LDs of which cryo-EM overview maps were acquired were used for this analysis, including lamellae which were not subjected to electron cryo-tomography (cryo-ET). **B - D)** HeLa cells expressing Cidec-EGFP (B), untagged Cidec (C) or not induced for expression of Cidec-EGFP (D) were vitrified, screened for LD-LD interfaces by cryo-FM, thinned by cryo-FIB milling and imaged by cryo-EM. The first and fourth panels from the left represent overview cryo-EM images of lamellae. Second and fifth panels correspond to a magnified image of the areas in blue boxes. Third panels represent virtual slices through tomograms acquired at areas where LDs are in close proximity indicated by the purple boxes in the second panel (B and C) or of an individual LD (D). Green arrows point to LD-LD interfaces. White arrows indicate individual LDs. Note that the second panel in B is from the same image as the fourth panel in Figure 1C. Scale bars 2.5 μm (first and fourth panels), 1 μm (second and fifth panels), and 150 nm (third panels).

Figure S4

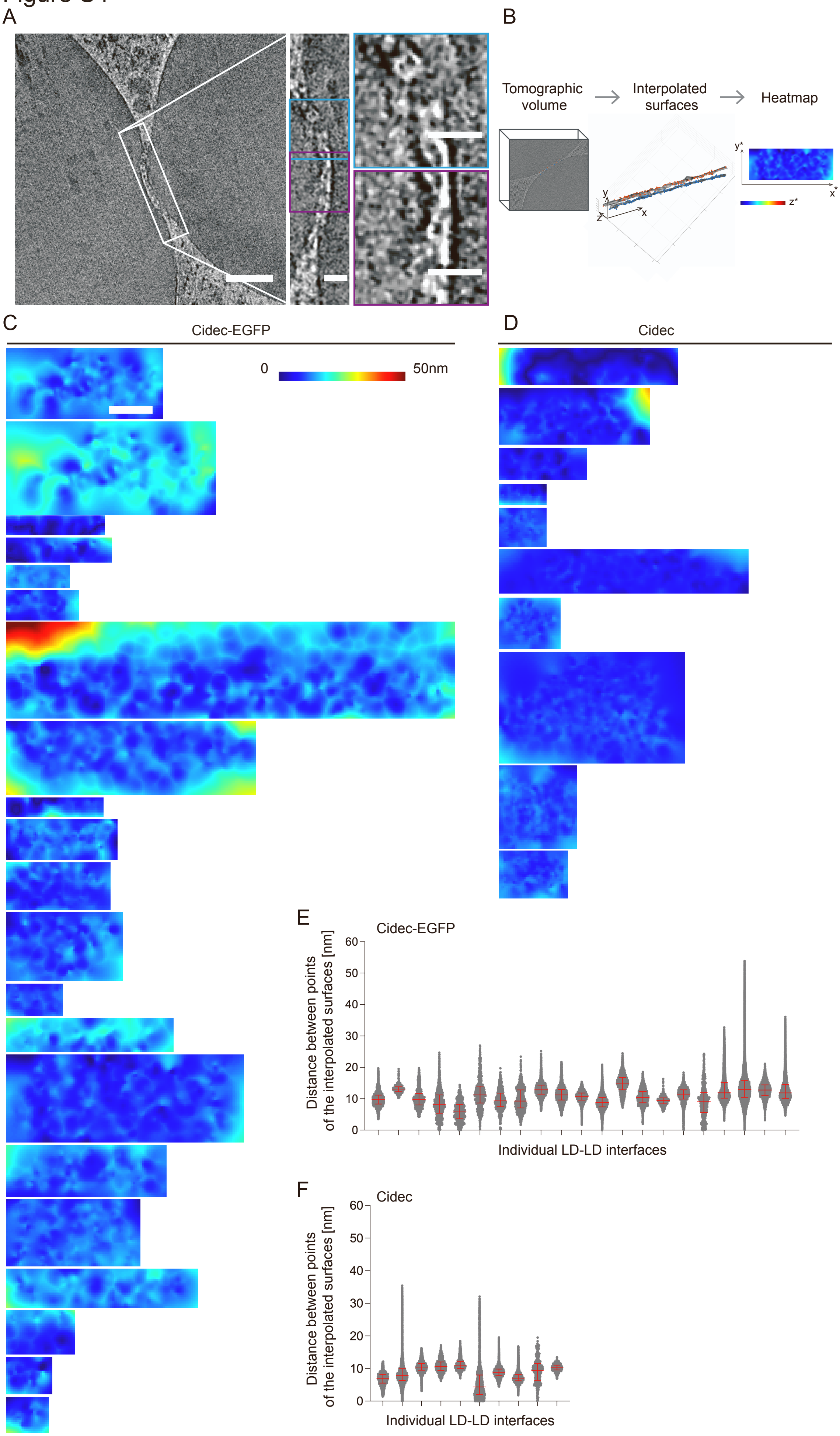
A
B
Tomographic volume
Interpolated surfaces
Heatmap
y*
x*
z*
y
x
z
C
Cidec-EGFP
0
50nm
D
Cidec
E
Cidec-EGFP
Distance between points of the interpolated surfaces [nm]
60
50
40
30
20
10
0
Individual LD-LD interfaces
F
Cidec
Distance between points of the interpolated surfaces [nm]
60
50
40
30
20
10
0
Individual LD-LD interfaces

Supplemental Figure S4: Details of LD-LD interfaces. Related to Figures 2 and 3. **A)** Virtual slices through a tomogram acquired at an area where LDs are in close proximity. HeLa cells inducibly expressing Cidec-EGFP were vitrified, screened for LD interfaces by cryo-FM, thinned by cryo-FIB milling and imaged by cryo-ET. The shape of the LDs is deformed where two LDs are closely apposed. The morphology displayed here shows a protrusion of one LD into the other LD, resulting in an indentation. The image is the same as shown in Figure 2C. The degree of deformation varies along the interface (white rectangle, magnified in second image): some areas display large deformation of the monolayers (blue square) and others slight waviness (purple square). Scale bars: 100 nm in large image (left panel) and 25 nm in magnified views. **B)** Schematic overview of distance measurements at LD interfaces. Points were picked manually along both monolayers in virtual tomographic slices. The monolayers were thereby traced through the entire tomographic volume (first panel). Based on the manually picked points, interpolated surfaces are computed as 2 nm-spaced regular grid points (middle panel). The distances between these grid points are represented as a heatmap, with x, y corresponding to the position of the regular grid (third panel). **C)** and **D)** Heat maps (generated as explained in B) illustrating the variability of distances between the monolayers at interface formed by Cidec-EGFP (C) and by untagged Cidec (D). Each panel corresponds to one individual interface. The scale bar in the top left panel corresponds to 50 nm and applies to all panels. The heat map legend also applies to all panels; dark blue corresponds to an inter-monolayer distance of 0 nm and dark red corresponds to an inter-monolayer distance of 50 nm. **E)** and **F)** The distances measured between the regularly spaced points of the interpolated surfaces plotted as scatter plots for each individual interface. The red lines correspond to medians and interquartile range. The medians are plotted in Figure 3F. **E)** Interfaces between LDs in cells expressing Cidec-EGFP. **F)** Interfaces between LDs in cells expressing untagged Cidec.

Figure S5

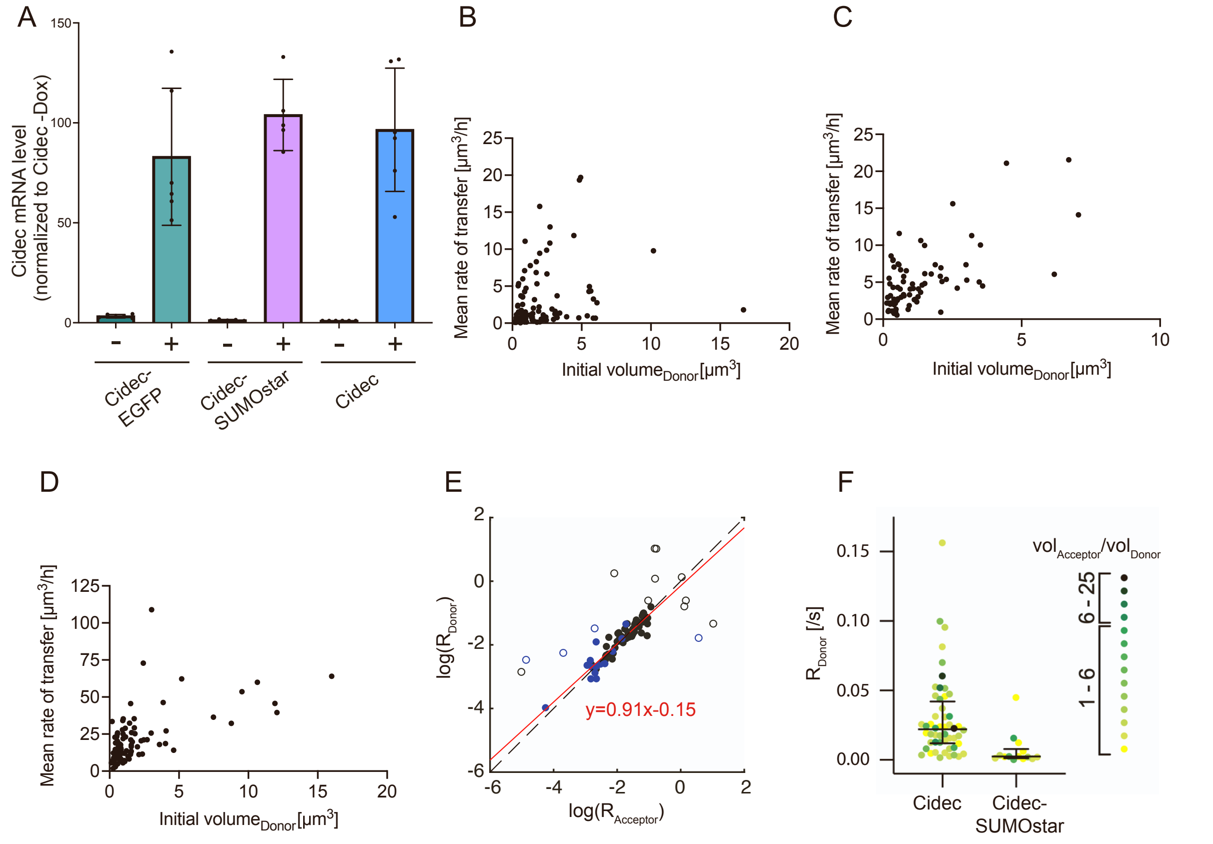

<u>Supplemental Figure S5: Characterisation of Cidec constructs.</u> Related to Figures 4 and 5. **A)** HeLa cell lines were induced with Doxycycline for expression of Cidec constructs in the presence of 200 μM oleic acid for 24 hours. Comparable levels of *Cidec* transcripts were achieved by using 0.5 μg/mL of Doxycycline in Cidec-EGFP and untagged Cidec HeLa cell lines and 2.0 μg/mL of Doxycycline in the Cidec-SUMOstar HeLa cell line. mRNA levels were normalized to the level of untagged Cidec HeLa cell line without Doxycycline treatment. The plot shows mean Cidec mRNA expression levels with SD from at least 5 independent experiments. **B-D)** The mean rates of transfer (same data as shown in Figure 4D) plotted against the initial volume of the donor LD measured at the start of the event, for Cidec-EGFP (B), Cidec-SUMOstar (C) and untagged Cidec (D). **E)** Detection of outliers. Log10 of R-values, calculated from exponential fits as shown in Figure 5C, D and E, of acceptor LD are plotted against log10 of R-values of the corresponding donor LD. Cidec: black circles. Cidec-SUMOstar: blue circles. Red equation describes regression line (shown in red). Black dashed line indicates y=x. Pairs differing by more than 0.4 (empty circles) were excluded from further analysis (for details see text and STAR Methods). **F)** R_{Donor} values for untagged Cidec (n=52) and Cidec-SUMOstar (n=14). This is the same data

as shown in Figure 5F, except that each data point, corresponding to one event, is color-coded according to the starting ratio of acceptor volume to donor volume. Black bars correspond to median and interquartile range (Cidec: n=52; median: 0.022 /s, Cidec-SUMOstar: n=14; median: 0.002 /s. $P<0.0001$).
